# Research Progress on Peptide Drugs for Type 2 Diabetes and the Possibility of Oral Administration

**DOI:** 10.3390/pharmaceutics16111353

**Published:** 2024-10-23

**Authors:** Xinxin Yang, Ruiting Lin, Changzhuo Feng, Qiyuan Kang, Peng Yu, Yongzhi Deng, Ye Jin

**Affiliations:** 1 School of Pharmacy, Changchun University of Chinese Medicine, Changchun 130117, China; yangxx@ccucm.edu.cn (X.Y.); 23203070208@stu.ccucm.edu.cn (R.L.); 2 College of Traditional Chinese Medicine, Changchun University of Chinese Medicine, Changchun 130117, China; 21203070201@ccucm.edu.cn (C.F.); 2302040121@ccucm.edu.cn (Q.K.); yupeng@ccucm.edu.cn (P.Y.)

**Keywords:** oral delivery, nano-formulations, peptide drugs, type 2 diabetes mellitus

## Abstract

Diabetes is a global disease that can lead to a range of complications. Currently, the treatment of type 2 diabetes focuses on oral hypoglycemic drugs and insulin analogues. Studies have shown that drugs such as oral metformin are useful in the treatment of diabetes but can limit the liver’s ability to release sugar. The development of glucose-lowering peptides has provided new options for the treatment of type 2 diabetes. Peptide drugs have low oral utilization due to their easy degradation, short half-life, and difficulty passing through the intestinal mucosa. Therefore, improving the oral utilization of peptide drugs remains an urgent problem. This paper reviews the research progress of peptide drugs in the treatment of diabetes mellitus and proposes that different types of nano-formulation carriers, such as liposomes, self-emulsifying drug delivery systems, and polymer particles, should be combined with peptide drugs for oral administration to improve their absorption in the gastrointestinal tract.

## 1. Introduction

Diabetes is one of the major causes of long-term ill health and a growing global disease. Often leading to severe metabolic disease and a range of serious complications, diabetes is a global disease burden [1]. Diabetes mellitus is classified as type 1 diabetes mellitus (T1DM) and type 2 diabetes mellitus (T2DM). T1DM is characterized by insufficient insulin secretion, which renders pancreatic islet cells incompetent against autoimmune [2]. T2DM is the most prevalent type, with a longer disease course, and is characterized by hyperglycemia due to a decrease in insulin secretion by the pancreas [3]. T2DM also causes complications such as cardiovascular disease and type 2 diabetic nephropathy, as well as an increased risk of hypertension [4,5]. T2DM is characterized by relative insulin deficiency or insulin resistance, in which the muscles and organs, such as the liver, are not sensitive enough to insulin so that blood glucose cannot be used effectively and remains at a high level [6]. In recent years, treatment for T2DM has focused on drugs that increase insulin secretion, sensitize, and reduce hepatic glucose production. Other non-modifiable risk factors include family history and ethnicity [7]. Obesity, disrupted sleep cycles or abnormal circadian rhythms, and a lack of sufficient regular physical activity may have compounding effects [8]. While current treatment strategies can help people with diabetes improve their symptoms, new and effective treatments are needed.

In recent years, peptide drugs synthesized using modern biotechnology have become one of the hotspots of drug research and development and are now widely used in the prevention, diagnosis, and treatment of tumors, hepatitis, diabetes, AIDS, and other diseases due to their wide indications, high safety, and remarkable efficacy, and have a broad development prospect. Some peptide drugs use less dosage, are more selective, have better specificity, and have fewer side effects than small-molecule drugs. Peptide therapeutics are usually administered by injection, but injections are invasive and reduce patient compliance. In addition, injectables increase the complexity and cost of manufacturing and storage. There is a growing interest in the oral delivery of peptides, a technology that could provide a safe and convenient alternative to peptide drug delivery [9]. However, direct delivery of peptides is not feasible. Oral peptides must overcome physiological barriers such as low solubility, permeability, and early degradation to achieve efficient and sustained drug delivery [10].

**Figure 1:**
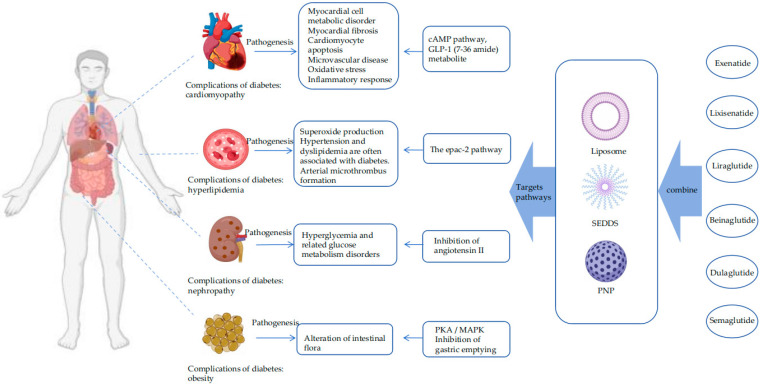
The mechanism/target of the combination of peptide drugs and nano-preparations on diabetic complications.

In exploring technological approaches to achieve efficient oral peptide delivery, drugs combined with nano-formulations are particularly promising due to their wide diversity, good biocompatibility, and ease of modification and thus functionalization [11]. As shown in Figure 1,this paper reviews the current research progress, therapeutic scope, and potential applications of peptide drugs for the treatment of diabetes (Table 1) and lists oral formulations that can be loaded with peptides. It is hoped that combining the two will provide new ideas for the oral delivery of peptide drugs for the treatment of diabetes.

## 2. Glucagon-like Peptide 1 (GLP-1)

**Figure 2:**
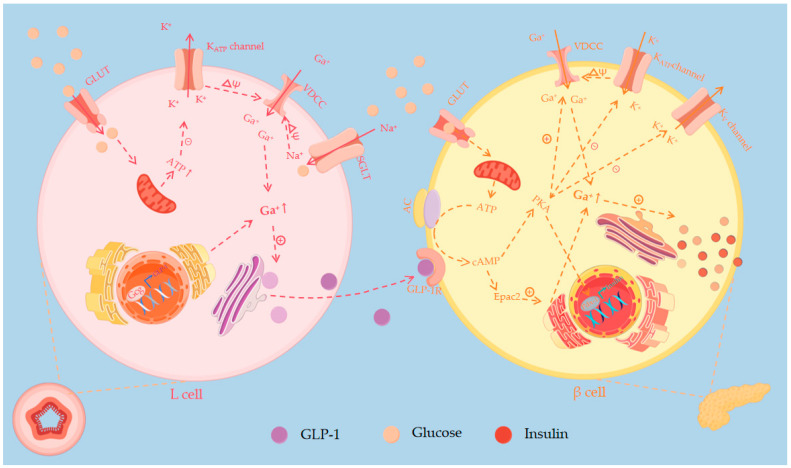
Glucose stimulates GLP-1 secretion from L-cells: Glucose closes ATP-sensitive K^+^_ATP_ channels and opens Ca^2+^ channel (VDCC) to induce membrane depolarization, and Ca^2+^ inward flow triggers vesicular cytotoxicity and GLP-1 secretion.

GLP-1 is a peptide derived from the *glucagonogen* (*Gcg*) sequence that shares the same precursor molecule as glucagon but has an opposite effect on blood glucose. GLP-1 is mainly secreted by intestinal L-cells and enters the peripheral circulation after entering the bloodstream, while glucagon preprogenitor neurons in the solitary tract nucleus of the brainstem also secrete GLP-1 and play a role in the central nervous system (CNS) [20,21]. Prohormone convertase 1 (PC1) cleaves *Gcg* in cells, thereby expressing GLP-1 [22,23,24]. GLP-1 promotes insulin secretion after binding to and activating the glucagon-like peptide-1 receptor (GLP-1R) on the surface of β-cells, and glucose dose-dependently increases the concentration of GLP-1 secreted by L cells. GLP-1 also inhibits β-cell apoptosis and induces β-cell proliferation by stimulating the expression of insulin receptor substrate 2, including mechanisms such as acute proinsulin action and stimulation of second messenger cAMP by activation of the response element binding protein (CREB) (Figure 2) [25,26]. Under conditions of insulin resistance and cellular stress, α-cells can regulate insulin secretion by producing GLP-1, thereby compensating for the increased functional demands of β-cells [27]. In addition, GLP-1 can stimulate growth inhibitor secretion and activate the protein kinase A system (PKA) to inhibit α-cell glucagon secretion [28]. Because GLP-1 is cleaved in vivo by dipeptidyl peptidase-4 (DPP-4) (EC number: 3.4.14.5) and the circulating half-life of GLP-1 in diabetic patients is only 1.5 to 2 min [29], GLP-1 receptor agonists (GLP-1RAs) for the treatment of T2DM are widely developed and used.

GLP-1R is expressed in the heart, brain, kidneys, stomach, and fat cells and ameliorates complications caused by diabetes. GLP-1R is mainly found in cardiac endothelium, coronary arteries, and smooth muscle cells [21,30]. GLP-1 is cardioprotective through the cAMP signaling pathway and DPP-4 degradation of GLP-1 (7–36 amide) to generate GLP-1 (9–36 amide) and its smaller degradation products. Thus, GLP-1RAs increase cardiomyocyte survival and improve endothelial dysfunction [31]. GLP-1 can improve blood pressure by stimulating urinary sodium excretion through epac2-dependent stimulation of atrial natriuretic peptide secretion [32]. Brain GLP-1R activation feeds through PKA/MAPK-induced AMPK inhibition. Peripheral GLP-1 is transmitted via the vagus nerve to the central nervous system to generate electrical signals, and brain-derived GLP-1 is released locally as a neurotransmitter, which together mediate inhibition of gastric motility and reduce the rate of glucose absorption [33,34]. In addition, GLP-1 inhibits gastric emptying through adrenergic signaling and promotes brown remodeling of white fat in a SIRT1-dependent manner, reducing body weight in T2DM patients [35,36,37]. GLP-1 inhibits angiotensin II and renin and reduces the risk of proteinuria in T2DM patients [38]. Although the exact details of renal regulation by GLP-1 are unknown, it has been found that GLP-1 ameliorates renal injury, fibrosis, and lipid accumulation in diabetic nephropathy rats via the Sirt1/AMPK/PGC1α pathway [39]. In addition, the researchers isolated a GLP-1RAs called Exendin-4 from lizard venom and optimized the structure of GLP-1 and Exendin-4 for the treatment of T2DM [40].

GLP-1 stimulates β cells to secrete insulin. GLP-1-induced elevation of cAMP leads to PKA activation and enhanced signaling via exchange proteins directly activated by cAMP (Epac). cAMP activation of PKA increases the sensitivity of the K^+^_ATP_ channel to ATP, leading to the closure of the K^+^_ATP_ channel, depolarization of the cell membrane, and opening of the VDCC channel. Subsequent Ca^2+^ influx promotes cytosolic action of insulin granules and acute secretion of insulin into the circulation. Simultaneously, GLP-1-activated PKA inhibits voltage-gated Kv channels, prevents membrane repolarization, and promotes Ca^2+^ influx by prolonging the opening of the VDCC channel. Epac stimulates the release of Ca^2+^ from the endoplasmic reticulum (ER), increasing insulin secretion by increasing the intracellular pool of Ca^2+^. cAMP activates Epac 2 by direct binding. Under high glucose conditions, the flow of Ca^2+^ through the VDCC channel into b-cells enhances Ca^2+^ influx, and Epac 2 opens ryanodine receptor Ca^2+^ channels in the ER, further increasing intracellular Ca^2+^ levels and enhancing insulin secretion.

### 2.1. Exenatide

In April 2005, Exenatide was approved by the US FDA as the first GLP-1RAs for the treatment of T2DM. Exenatide has 50% homology to human GLP-1 and similar affinity for the GLP-1 receptor. It exhibits glucose-regulatory activity similar to that of GLP-1, including enhanced glucose dependence of β-cells in response to insulin secretion, and is accompanied by progressive weight loss. Exenatide reduces the concentration of glucagon in the blood during periods of hyperglycemia. In patients with T2DM, glucagon levels are abnormally high during hyperglycemia, and lowering blood glucagon concentrations during hyperglycemia leads to reduced hepatic glucose output and reduced insulin requirements [41]. In addition, exenatide may also reduce renal dysfunction to a certain extent in patients with diabetic nephropathy, with exenatide significantly reducing proteinuria levels in 26.2% of the patients [42]. Exenatide mediates attenuation of diabetic nephropathy by SIRT1-dependent down-regulation of thioredoxin-interacting protein H3K9ac acetylation and reduces the recruitment of spliced X-box binding protein 1 to the Txnip promoter [43]. Weekly doses of exenatide have also been shown to modestly reduce body weight and systolic blood pressure [44]. Exenatide administered at a dose of 2 mg once a week (QW) showed greater improvements in glycemic control compared to the 10 µg dose taken twice a day (BID), with no increased risk of hypoglycemia and a corresponding reduction in body weight [45]. Compared with other hypoglycemic agents, exenatide significantly improved carotid intima-media thickening, which is associated with cardiovascular probability, and its cardioprotective effects were demonstrated by the inhibition of atrial fibrillation and reduced susceptibility to atrial fibrillation in rats [46,47]. Gastrointestinal adverse events (AEs), including nausea, vomiting, and diarrhea, are common with exenatide during treatment. In 2021, Bydureon, whose main ingredient is exenatide, became the first FDA-approved microsphere formulation for the treatment of T2DM, attenuating gastrointestinal AE. However, subcutaneous nodules can occur at the injection site when the microspheres produce an inflammatory foreign-body reaction in patients treated with exenatide QW [48]. Injection site reactions were more common in the exenatide QW group than in the exenatide BID or non-GLP-1RAs groups. In addition, after the initial release of surface-bound exenatide, the encapsulated peptide was released from the microspheres in approximately two weeks’ time [49].

### 2.2. Loxenatide

Loxenatide, also known as polyethylene glycol loxenatide (PEX-168). It is based on the structure of exenatide and modifies the drug molecule by adding polyethylene glycol, thus extending the half-life of the drug [40]. Clinical trials showed that loxenatide monotherapy significantly improved glycemic control in patients with T2DM, with a safety profile similar to other GLP-RA [14]. A short-term controlled trial with metformin showed that the addition of loxenatide to metformin was superior to the addition of glucagon in improving glycemic control and assessing glycemic variability with continuous glucose monitoring in patients with T2DM [14]. Weight loss was more pronounced in patients given loxenatide subcutaneously once a week compared to metformin, and loxenatide reduced body weight by no more than 20% in purely obese mice irrespective of dose, with some risk of hypoglycemia [50,51]. T2DM can lead to impaired endothelium-dependent vasodilatation and decreased NO expression, manifesting as cardiovascular and endothelial dysfunction. The T2DM patient population with loxenatide showed significant improvements in glycemic lowering indices and inflammation-related indices, as well as significant changes in vascular endothelial cell function-related indices [52]. A controlled trial also demonstrated that loxenatide improved endothelial cell function in patients with T2DM [53]. Loxenatide reduces high glucose-induced ROS production in a concentration-dependent mechanism and inhibits mitochondria-dependent apoptosis of endothelial progenitor cells (EPCs) and mitochondrial membrane depolarization via activation of the sirtuin 3/Foxo3 signaling pathway and enhances mitochondrial respiratory function [54].

### 2.3. Lixisenatide

Lixisenatide, an Exendin-4 analogue, was approved in the EMA in 2012 for the treatment of T2DM. In preclinical studies, lixisenatide demonstrated high affinity and selectivity for the GLP-1 receptor, binding approximately four times that of natural GLP-1 [55]. Clinical studies have evaluated the efficacy of lixisenatide in combination with oral hypoglycemic agents in the treatment of patients with T2DM, and changes in glycated hemoglobin (HbA1c) demonstrated that the combination of lixisenatide with oral hypoglycemic agents significantly improves the efficacy and safety profile [56]. In an analysis of the results of a phase 3 clinical trial, it was reported that the majority of patients receiving a fixed ratio of glycemic insulin/lixisenatide were no longer experiencing gastrointestinal AE at eight weeks, with a greater potential to achieve target blood glucose compared to those receiving lixisenatide alone [57]. Lixisenatide stimulates insulin secretion when blood glucose is elevated but not when blood glucose is normal, thereby circumventing the risk of hypoglycemia and glucose-dependently inhibiting glucagon secretion [58,59]. The major differences from other GLP-1 RAs are that lixisenatide significantly reduces postprandial glucose immediately after injection and that the delay in gastric emptying is more pronounced with lixisenatide. The increase in intragastric glucose residence time after lixisenatide treatment was associated with a decrease in the systemic oral glucose rate and a decrease in the area under the glycemic curve, and this effect was more pronounced as the duration of dosing increased [60]. Lixisenatide also improves endothelial cell function and inflammation-related markers in T2DM patients [61]. Lixisenatide carries out anti-inflammatory effects by inhibiting the activation of NF-kB signaling in HUVECs cells and inhibiting the adhesion of monocytes and HUVECs [62]. and promotes mitochondrial biogenesis in HUVECs by inducing phosphorylation at the Ser133 site of CREB [63]. Lixisenatide is filtered in the kidney and subsequently degraded in the renal tubules [64]. Lixisenatide dose adjustment is not required in patients with T2DM with mild or moderate renal impairment [65].

### 2.4. Liraglutide

Liraglutide is based on the natural GLP-1 structure and is 97% homologous to natural GLP-1 [40], chemically modified to extend its half-life to 13 h after subcutaneous injection and to resist DPP-4 degradation [66]. Liraglutide was approved by the FDA in 2010 to improve glycemic control in adult patients with T2DM, and liraglutide is the only GLP-RAs approved for weight loss [67]. Clinical Trial Demonstrates Effectiveness of liraglutide as Monotherapy and Add-on to First-Line Therapy [49]. Liraglutide inhibits oxidative stress and reduces cardiovascular disease while controlling blood glucose [68]. Liraglutide elevated SIRT1 expression, which increased Parkin expression, leading to mitochondrial autophagy activation. Protective mitochondrial autophagy reverses cytosolic adenosine 5′-triphosphate production, reduces cellular oxidative stress and balances redox reactions, maintains mitochondrial homeostasis, and repairs infarcted hearts [69]. Liraglutide inhibits oxidative stress and apoptosis by increasing the concentration of anti-apoptotic proteins and decreasing the levels of pro-apoptotic proteins. Liraglutide treatment is confirmed to reverse microvascular endothelial cell injury [70]. Whereas liraglutide alone reduced pro-inflammatory and pro-atherosclerotic chemokines [71], liraglutide effectively counteracts high glucose-induced dysfunction of HUVECs by inhibiting PINK1/Parkin-dependent mitochondrial autophagy [72]. Together, they validate the vaso-protective effects of liraglutide. In both T2DM and non-T2DM patients, liraglutide reduces the onset and progression of renal disease in addition to conventional therapy [73,74,75]. Liraglutide protects renal thylakoid cells from hyperglycemia-mediated mitochondrial apoptosis by upregulating SIRT3 expression and activating the ERK-Yap signaling pathway [76]. Liraglutide also inhibits renal cell apoptosis by increasing SIRT1 expression and down-regulating TXNIP expression [72,77]. In addition, liraglutide reduces higher circulating plasma concentrations of dihydroceramide and ceramide, which are positively associated with insulin resistance and hepatic steatosis, in patients with T2DM [13].

### 2.5. Beinaglutide

Beinaglutide is an amine-free recombinant human GLP-1 (7–36) acid with 100% homology to human GLP-1. It is approved for the treatment of T2DM patients in China [78]. Beinaglutide shows significant weight loss benefits and effective glycemic control in the treatment of T2DM [79] and is more effective than metformin in reducing body weight and fat mass [80,81]. Although safe and effective in reducing visceral fat and body weight in obese T2DM patients, the dose of beinaglutide should be tailored to the individual [82]. Beinaglutide combats high-fat food-induced obesity by targeting the composition of major lipids in adipose tissue and the expression of lipid metabolism genes, e.g., by increasing Acrp30 levels and Akt phosphorylation in adipocytes, thereby ameliorating the associated fatty liver and dyslipidaemia [83]. In addition, serpin E1, leptin, c-reactive protein (CRP), and tumor necrosis factor-alpha (TNF-alpha) were significantly decreased in T2DM patients using beinaglutide, demonstrating that beinaglutide also has an anti-inflammatory effect in T2DM patients [84]. In a model of non-alcoholic steatohepatitis, long-term use of beinaglutide reduced liver weight and hepatic steatosis and improved insulin sensitivity, and increased Sirt1 gene expression levels improved hepatic insulin resistance [78].

### 2.6. Dulaglutide

Dulaglutide is produced by linking human immunoglobulin IgG-4 heavy chain to GLP-1 modification, which significantly increases its half-life [85]. In 2014, Dulaglutide became the first fragment crystallizable fusion protein to receive FDA approval for T2DM treatment [49]. Dulaglutide results in clinically dose-related reductions in HbA1c and body weight. In the six completed AWARD results, the majority of dulaglutide patients had HbA1c targets <7.0% (53.0 mmol/mol) and ≤6.5% (47.5 mmol/mol), which were superior to the active comparators (Exenatide, Glycine Insulin, Metformin, and Sitagliptin), with the most common adverse events being gastrointestinal AE, the lower rates of hypoglycemia [86,87,88]. In mouse control experiments, the expression levels of pancreatic beta-cell-related genes, insulin content, and insulin secretion were found to be significantly elevated in the dulaglutide group, and oxidative and endoplasmic reticulum stress, inflammation, fibrosis, and apoptosis were inhibited after treatment. Demonstration of a long-term protective effect of dulaglutide on β-cells [89]. In addition, dulaglutide can inhibit inflammation and oxidative stress by inhibiting the NF-κB signaling pathway and TGF-β/Smad2 signaling pathway, M1 macrophage polarization, and mitochondrial ROS release [90,91,92]. T2DM patients treated with dulaglutide improved systolic and pulse pressure, although one-third was due to weight loss effects [93]. However, the risk-related indices of urinary albumin/creatinine and HbA1c were reduced by 16.7% and 25.4%, respectively, in patients with adverse cardiovascular events with dulaglutide compared with placebo [94,95]. Dulaglutide may be considered for use in middle-aged and elderly T2DM patients with cardiovascular disease or cardiovascular risk factors [96]. Dulaglutide inhibits high glucose-induced activation of the NLRP3 inflammasome in HUVECs cells by increasing SIRT1 expression and suppressing NLRP3, ASC, and P10 expression. It also attenuated high glucose-induced cellular mitochondrial division in EPCs and significantly ameliorated vascular endothelial tissue damage and inflammation [97,98].

### 2.7. Semaglutide

Semaglutide is the first GLP-1RAs to have an oral dosage form. Semaglutide is prepared as a tablet in combination with the absorption enhancer sodium N-[8(2-hydroxybenzoyl)amino]octanoate (SNAC) to improve glycemic control in adults with T2DM [99,100]. SNAC binds to semaglutide, elevates local pH to decrease the efficacy of major digestive enzymes, increases the lipophilicity of semaglutide to promote gastric epithelial cell uptake, and facilitates the transit of semaglutide and its entry into the systemic circulation. When semaglutide and SNAC reach the bloodstream, the two molecules readily dissociate, allowing the oral formulation of semaglutide to interact with the body in the same manner as subcutaneous semaglutide [101]. However, the oral formulation of semaglutide has lower bioavailability compared to the subcutaneous formulation. Semaglutide is absorbed in the stomach with a rapid and dose-dependent increase in plasma concentrations, reaching peak concentrations within 15–35 min [102]. Semaglutide oral formulations at doses ≤ 10 mg are significantly less effective than subcutaneously administered forms, and therefore higher doses of semaglutide oral formulations need to be given to achieve the same clinical outcomes as subcutaneous formulations. Because SNAC protects semaglutide from pH-dependent degradation and because eating can lead to an increase in gastric pH, patients should fast prior to dosing and avoid eating for at least 30 min after dosing for optimal results [103,104].

Semaglutide is a favorable option for the treatment of T2DM, both subcutaneously and orally, and several clinical trials have demonstrated the safety and efficacy of semaglutide in the treatment of T2DM [105,106,107,108]. Semaglutide has shown significant efficacy in reducing HbA1c and body weight compared to other GLP-1RAs, although semaglutide can cause mild and transient gastrointestinal disturbances that decrease with continuation of treatment and increase the risk of cholelithiasis [109,110,111]. In controlled trials, 85% of participants taking oral semaglutide lost at least 5% of their body weight and had sustained improvements in obesity-related abnormalities such as hyperglycemia, hypertension, elevated lipids, and high-sensitivity c-reactive protein [112]. And semaglutide has been shown to be effective, safe, and well tolerated in T2DM patients with overweight and atherosclerotic cardiovascular disease [113]. Treatment with semaglutide Significantly Reduces Symptoms and Physical Limitations Associated with Obesity in Heart Failure Patients, Improves Exercise Function, and Reduces Weight [114,115]. Semaglutide ameliorates mitochondrial function and cardiac injury by modulating the PI3K-AKT signaling pathway [116]. Semaglutide avoids oxidative stress and apoptosis in the heart of diabetic mice by activating the Sirt1/AMPK pathway [117]. In a mouse model of HFpEF induced by obesity and T2DM, semaglutide improved cardiometabolic silhouette, cardiac structure, and cardiac function compared to weight loss induced by paired feeding. Transcriptomic and proteomic analyses showed that semaglutide improved left ventricular cytoskeletal function and endothelial function and restored protective immune responses in visceral adipose tissue. Exceeding the weight loss effect of paired feeding [118,119]. Semaglutide is Equally Effective in Glycemic Control and Weight in Patients with Chronic Kidney Disease [120]. Semaglutide directly ameliorates oxidative stress and inflammation-related metabolites such as NAD+ and adenosine in the kidneys of obese mice [121]. In a mouse model of hypertension-accelerated diabetic nephropathy, semaglutide significantly reduced hyperglycemia, hypertension, and proteinuria while significantly improving the severity of glomerulosclerosis and urine/kidney injury molecule-1 (KIM-1) levels [122].

## 3. Tirzepatide

2022 FDA approval of dual agonists for GLP-1R and glucose-dependent insulinotropic polypeptide receptor (GIPR) tirzepatide to improve glycemic control in adults with T2DM as an adjunct to diet and exercise [123]. Bringing renewed attention to the potential of glucose-dependent insulinotropic polypeptide (GIP) in the treatment of T2DM. GIP is a polypeptide consisting of 42 amino acids, which, along with GLP-1, belongs to the same group of enteric insulin in the human body and is secreted by the K cells of the intestinal mucosa of the duodenum and jejunum in the small intestine. GIP is responsible for most of the body’s enteric insulin action; a normal human postprandial GIP level is about 4 times that of GLP-1, which is the most important enteric insulin [124]. Although both GIPR and GLP-1 R are present in β-cells, impaired β-cell function in patients with T2DM affects the regulatory functions of GIP and GLP-1 [125]. In T2DM, GIP has a limited ability to stimulate insulin secretion during hyperglycemia and does not significantly reduce blood glucose concentrations, whereas GLP-1 still significantly regulates blood glucose concentrations in patients with T2DM [126]. Unlike GLP-1, GIP has both glucagon and insulinotropic effects in a glucose-dependent manner, stimulating glucagon secretion under hypoglycemic conditions and insulin secretion under hyperglycemic conditions [127]. GIPRs were abundantly distributed in adipose tissue, and most of the distribution regions of GIPRs and GLP-1Rs in the CNS did not overlap. GIP is involved in the metabolism of adipose tissue carbohydrates and lipids, and it increases lipid storage in white adipose tissue and decreases ectopic storage of fat in the muscle [128]. In contrast, GLP-1 indirectly promotes lipolysis, works with GIP to maintain healthy adipocytes, and increases adipocyte production and secretion of lipocalin to improve insulin sensitivity [129]. GLP-1 synergizes with GIP to promote metabolic homeostasis, prevent hyperglycemia and hypoglycemia, attenuate dyslipidemia, and reduce the risk of cardiovascular disease in patients with T2DM and obesity.

Tirzepatide was injected once weekly, and the pharmacokinetics of tirzepatide were dose-proportional over the dose range studied [130]. In all SURPASS trials, tirzepatide showed a dose-dependent decrease in HbA 1c regardless of background glucose-lowering therapy [19]. The glycemic efficacy of tirzepatide in the treatment of T2DM was found to be due to simultaneous improvements in key components of diabetes pathophysiology, including beta-cell function, insulin sensitivity, and glucagon secretion, in Phase 1 clinical trials [131]. Tirzepatide synergistically activates both GIPR and GLP-1R in T2DM patients. In in vitro studies, tirzepatide elicited significantly higher cAMP responses in human pancreatic beta cell lines than those observed with GLP-1 or GIP alone, and in isolated islets, tirzepatide overcame the effects of GLP-1R to induce a robust increase in glucagon secretion [130,132]. However, the relative activity of tirzepatide at GLP-1 R and GIPR may vary in subjects with and without diabetes and with the degree of hyperglycemia. In SURPASS-2, tirzepatide was associated with dose-dependent weight loss from baseline, with up to 65%, 48%, and 31% of subjects treated with tirzepatide 15 mg/week losing ≥10%, ≥15%, and ≥20% of their body weight, respectively [133]. Tirzepatide provided more significant weight loss compared to selective GLP-1 RA treatment. Preclinical studies demonstrated greater weight loss in obese mice administered tirzepatide than in mice administered selective GLP-1 RA. GIP enhances GLP-1-induced food intake and weight loss by augmenting melanocortin expression and neuronal activation in different neuronal populations in the hypothalamus; thus, co-activation of GIPR and GLP-1R in the brain enhances weight loss efficacy [134,135]. T Therapeutic efficacy and side effects of tirzepatide are similar to those of selective GLP-1RA, with common AEs being gastrointestinal reactions. Tirzepatide effectively slowed the decline in glomerular filtration rate, lowered the urinary albumin/creatinine ratio, and reduced circulating levels of several biomarkers associated with cardiovascular risk, improving cardiovascular risk [136,137].

## 4. Oral Medications for Type 2 Diabetes

Metformin is considered the best initial therapeutic agent for T2DM. Regarding the mechanism of action of this drug, it may be related to the reduction in hepatic glucose production [125]. In randomized trials of diabetes, metformin was most effective in young, obese subjects. The results showed a decreasing risk of diabetes and long-term weight loss in the subjects [126]. Glipizide, a second-generation sulfonylurea, is a gastrointestinal therapeutic system (GITS) extended-release formulation. Glipizide GITS provides more stable plasma drug concentrations than immediate-release formulations, and the once-daily dosing regimen optimizes patient compliance. In patients with T2DM, the effect of glipizide GITS on fasting blood glucose levels may be greater. Glipizide GITS was shown to reduce the incidence of hypoglycemic symptoms in a 3-year model of type 2 diabetes treatment [123]. Oral repaglinide is a rapid-acting insulinotropic agent that reduces postprandial glucose by targeting early insulin release, and lowering postprandial glucose is considered to be an important factor in the reduction in long-term cardiovascular complications of diabetes. It has a unique binding site on the beta cell membrane, and in in vitro and in vivo studies, alogliptin has been shown to be more potent and faster-acting in terms of insulinotropic effects, but may cause some weight gain [122]. DPP-4 inhibitors are widely used in the treatment of T2DM, which results in intra-insulin release when blood glucose is elevated. After evaluating the patients, the results suggest that DPP-4 inhibitors promote vascular endothelial cell regeneration, but when this effect occurs in the glomerulus, the proliferation of glomerular endothelial cells leads to tma-like lesions, which cause increased proteinuria and a rapid decline in renal function [124]. Miglitol is a second-generation alpha-glucosidase inhibitor that is generally well tolerated and does not cause weight gain or hypoglycemia with monotherapy. The drug is systemically absorbed but not metabolized and is rapidly excreted through the kidneys. Most of the adverse reactions associated with miglitol therapy involve gastrointestinal AE [127]. Pioglitazone is a thiazolidinedione drug that selectively agonizes the peroxisome growth factor-activated receptor PPARγ, which enhances insulin sensitivity in peripheral tissues and the liver and reduces insulin resistance. Pioglitazone significantly improved beta-cell function, liver function, and blood glucose indexes and slowed down the process of atherosclerosis. However, patients taking pioglitazone medication at high doses and for long periods of time may have an increased risk of bladder cancer [138]. Dagliflozin is a sodium-glucose cotransporter protein 2 (SGLT2) inhibitor that lowers blood glucose by inhibiting SGLT-2, reducing glucose reabsorption, and excreting sugar directly from the urine. In addition, dagliflozin can also reduce sodium reabsorption so that plasma volume decreases, achieving an antihypertensive effect and cardiovascular protection. And it reduces blood uric acid and proteinuria, relieves glomerular pressure, restores glomerular filtration rate, and controls the development of diabetic nephropathy [139] (Table 2).

Metformin is currently the first-line hypoglycemic agent of choice for the treatment of diabetes, either alone or as a base agent in various combination regimens, and almost half of patients with T2DM require additional hypoglycemic agents within 1 year of diagnosis. When considering second-line therapy, the central decisions facing providers and patients include safety, efficacy, tolerability, ease of administration, and cost. Several current second-line therapies, including sulfonylureas, glinides, DPP-4 inhibitors, alpha-glucosidase inhibitors, thiazolidinediones, SGLT-2 inhibitors, insulin, and GLP-1RA. Sulfonylureas, thiazolidinediones, and insulin can significantly improve glycemic control, but all three can cause weight gain, and sulfonylureas and insulin analogs can also cause a high risk of hypoglycemia. DPP-4 inhibitors, on the other hand, are less risky but lag behind insulin, sulfonylureas, and thiazolidinediones in glycemic control and are costly. Similar to DPP-4 inhibitors, SGLT-2 inhibitors have been associated with a low risk of hypoglycemia and high costs. However, unlike weight-neutral DPP-4 inhibitors, SGLT-2 inhibitors result in weight loss and lower blood pressure, and some members of this class have specific cardiovascular indications.

The advantage of GLP-1RA is that it has glucose-lowering efficacy similar to that of insulin but without the side effect of weight gain that accompanies insulin therapy. GLP-1RA also provides weight loss and a lower risk of hypoglycemia and has cardiovascular protective properties. Oral small-molecule drugs for GLP-1RA are currently under development and may further alter the use of glucose-lowering drugs.

Orforglipron is a non-peptide oral GLP-1RA biased toward G-protein activation. Orforglipron achieved similar blood glucose-lowering ability to exenatide at low blood concentrations in animal studies, and its G-protein bias may play a role in therapy [145]. Results from phase 2 clinical trials showed that orforglipron at doses of 12 mg or higher significantly reduced HbA1c and body weight compared to placebo or dulaglutide, with AEs similar to other GLP-1RAs [146]. Danuglipron, an oral small molecule GLP-1RA, demonstrated safety with twice-daily danuglipron in an 8-week randomized, double-blind trial, and 24-h mean daily glucose and HbA1c increased proportionally to the danuglipron dose [147]. In clinical trials, danuglipron demonstrated similar glucose-lowering effects to GLP-1RA and slightly reduced blood pressure in patients [148,149]. HRS-7535 is a novel oral small molecule GLP-1RA that significantly improves glucose tolerance, promotes insulin secretion, and reduces food intake without strict food restriction. In phase 1 trials, the pharmacokinetics of HRS-7535 were roughly proportional to the dose, significantly reducing patients’ body weight, with nausea and vomiting as common AEs, and its safety has been demonstrated [150]. There are other GLP-1RAs in other stages of development, e.g., Teng Ma et al. synthesized a small-molecule peptide that is a dual agonist of GLP-1R and GIP. It acts similarly to GIP and exendin-4 in a dose-dependent manner. When co-administered with sodium deoxycholate (SDC), SDC increased its bioavailability, and its oral hypoglycemic activity was enhanced. It also significantly improved glucose tolerance and hepatic fibrosis area in mice [151].

## 5. Oral Strategies for Peptide Drugs

**Figure 3:**
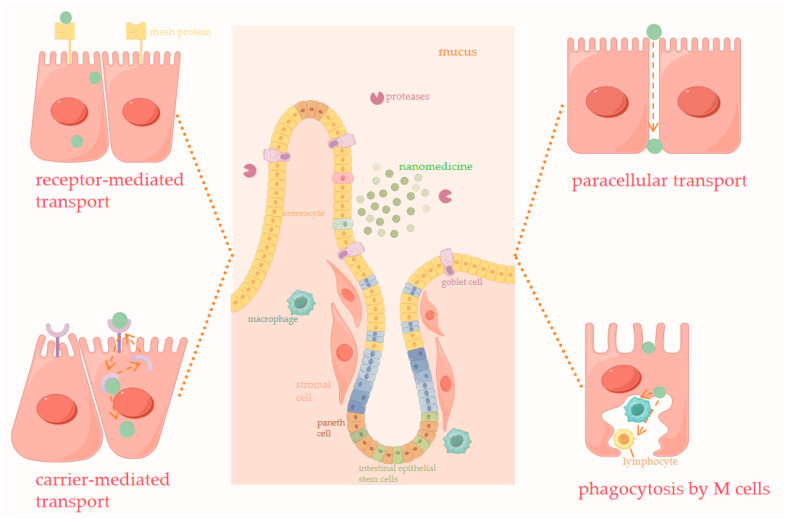
Schematic representation of the transport mechanisms.

Oral administration holds better promise for the treatment of T2DM than injectable administration. The main limitations of oral delivery of peptides are how to avoid degradation in the digestive system and improve bioavailability. Both the PH environment and digestive enzymes in the gastrointestinal tract greatly reduce the bioactivity of peptides in the GI tract, and the mucus layer and intestinal epithelium in the intestine are major barriers to the entry of peptides into the somatic circulation. Although semaglutide has been successfully prepared for oral administration, this customized approach cannot be directly replicated by other GLP-1Rs. In contrast, nanocarriers can be prepared into oral formulations without altering the structure of GLP-1R, improving bioavailability and prolonging in vivo circulation time through different absorption routes (Figure 3).

### 5.1. Liposome

Liposomes are spherical vesicles composed of phospholipid bilayers with biological properties similar to those of cell membranes. Intestinal epithelial cells are tightly packed with protein complexes that limit intercellular transport of macromolecules, so liposomes with a structure similar to that of the cell membrane can mediate cellular transport via receptors [152]. In addition, by changing the physical or chemical properties of the liposome surface and thus the nature of the vesicles, different purposes of delivery can be achieved [153,154].

The mucus layer covering the surface of the intestinal epithelial cells is the first physical barrier that prevents drugs from entering the body circulation, rapidly trapping and eliminating larger molecules and pathogens through adhesion, protecting the intestinal tract from pathogen invasion whilst restricting the diffusion of drug absorption. The mucus layer is mainly composed of highly glycosylated mucins, which consist of a protein backbone and oligosaccharide side chains, and the mucus adheres to particles, especially those with positively charged or hydrophobic surfaces, mainly through multivalent adhesion interactions generated by the carboxyl groups of the oligosaccharides and the negative charge of sulphuric acid [155]. Wang and colleagues designed protein-crowned liposomes for insulin delivery by covering cationic liposomes with bovine serum albumin (BSA). Proteins can be adsorbed on the surface of nanoparticles in biological fluids to form a protein envelope, which is known as a protein crown. Hydrophilic and neutrally charged protein crowns promote mucus permeation of protein-crowned liposomes. As protein-crowned liposomes permeate the intestinal mucus, the protein crown is progressively degraded by enzymes, and cationic liposomes interact with anionic cell membranes to achieve trans-epithelial transport of insulin. The results showed that protein-crowned liposomes had an oral bioavailability of up to 11.9% and were effective in lowering blood glucose levels [156]. Ding and colleagues utilized electrostatic interaction forces to cause BSA to form protein crowns on the surface of liposomes and bind to cell-penetrating peptides to load liraglutide, which was used to improve the mucus permeability of the liposomes and enhance their absorption efficiency in the gut. In vitro experiments demonstrated the stability of liraglutide after liposome release. In vivo experiments have demonstrated that liposomes loaded with liraglutide have good intestinal endocytosis, a long intestinal absorption time, and good biosafety [157].

Recombinant microorganisms can be used as natural liposomes loaded with peptides and have unique advantages in biocompatibility. Recombinant microbial drug delivery avoids stimulation by environmental exogenous substances and controls the release of drugs or prodrugs in the body, and specific gene editing of recombinant microbial discourse specifically regulates homeostasis [158]. Fei et al. recombined and loaded *Rhodotorula glutinis* with peptides and insulin for oral administration to Type 2 diabetic mice. The cells of the recombinant strain accumulated more lecithin and fatty acids, which contributed to the loading of the peptide drug. Recombinant strain cells no longer proliferate in mice and slowly release the loaded drug; different levels of insulin were found in serum and tissues and significantly reduced blood glucose levels in mice [159]. Oral drug delivery based on recombinant microorganisms holds great promise, but some of the absorption mechanisms still require in-depth study.

Exosomes are external vesicles secreted by cells that can be released by all kinds of cells, such as human exosomes, plant exosomes, and animal exosomes. Exosomes have low immunogenicity and toxicity and offer advantages in the targeted delivery of therapeutic molecules because of the specific interactions between the surface proteins of these exosomes of different cellular origins and their target cells [160]. Currently the most promising of the oral exosomes are the milk-derived exosomes, which can promote the proliferation of intestinal epithelial cells, enhance intestinal permeability, and have the ability of immunomodulation [161]. Wu et al. prepared milk exosomes and loaded them with insulin for the treatment of Type 1 diabetic mice. Bovine milk exosomes were found to be actively multi-targeted to epithelial cells through active transport, membrane fusion, and niche-mediated transport. In addition, cow’s milk exosomes can regulate surface pH during transport to adapt to different environments, avoid endolysosomal restriction, and promote uptake and cytosolization. And subcutaneous insulin has a more sustained glycemic-lowering effect than oral milk exosome-loaded insulin [162]. Xiao and colleagues, on the other hand, utilized the flow and fusion properties of the phospholipid bilayer of the exosome membrane to fuse milk exosomes with modified liposomes. A hybridized vesicle with adaptive surface properties loaded with semaglutide for oral delivery was prepared. Hybridized vesicles contain about 85% of exosomal membrane proteins but encapsulate semaglutide at 2.4 times the rate of natural milk exosomes and are much more hydrophilic, while modified liposomes give hybridized vesicles a neutral surface and are pH-responsive and self-regulating. Hydrophilicity and neutral charge facilitate penetration of the intestinal mucus barrier, and upon reaching the surface of jejunal epithelial cells, the highly retained membrane proteins and positively charged mixed vesicle surfaces effectively overcome the apical barrier, the intracellular transit barrier, and the basolateral cytosolic barrier. Adaptive surface characterization of hybrid vesicles increased the oral bioavailability of semaglutide to 8.7%, significantly improving the pharmacological therapeutic effect [163]. Lactogenic exosomes are widely used in oral delivery, and exosome delivery studies of macromolecules are about to become hot research.

### 5.2. Self-Emulsifying Drug Delivery Systems (SEDDS)

SEDDS are liquid or solid formulations consisting of an oil phase, a nonionic surfactant, and a co-surfactant, which can spontaneously emulsify into emulsions with particle sizes around 100–500 nm by gentle agitation in the gastrointestinal tract or at room temperature. SEDDS are lipid-based formulations that pair hydrophilic molecules such as GLP-1R and proteins with hydrophobic ions of oppositely charged lipophilic auxiliaries to increase their hydrophobicity and improve lipid solubility and can be incorporated into SEDDS for oral administration.

Gastrointestinal hydrolytic enzymes such as pepsin, trypsin, and other peptidases and proteases are insoluble in oily SEDDS droplets but can degrade esters such as triglycerides in SEDDS; therefore, SEDDS loaded with GLP-1R should use a lipolytic stabilizing component to avoid enzymatic degradation. The addition of mucus pro-osmotic agents to SEDDS and a reduction in the size of SEDDS droplets can increase the rate of contact between SEDDS and epithelial cells and improve the amount of drug entering the corpuscular circulation [164,165]. Claudia and coworkers prepared ORAL SEDDS for Exenatide, where a solution of Exenatide was shock-mixed with a solution of the oppositely charged hydrophobic ion docusate, which was paired lipidated and added to the SEDDS. Exenatide interacts with docusate only by inter-ionic forces without structural changes, ensuring the stability and pharmacological effects of exenatide. SEDDS consists of Labrafil 1944, which lacks a triglyceride structure, and Capmul PG-8, whose high HLB value gives SEDDS a smaller droplet size and better mucus permeability. In healthy rats, the SEDDS bioavailability of loaded exenatide was approximately 9% and sustained release for at least 6 h. Healthy rats exhibit better mucus permeability and lower blood glucose levels in vivo, indicating the oral potential of peptides paired with hydrophobic ions in SEDDS [166]. Another hydrophobic ion pairing, n-octadecyl sulfate, was found to pair better with exenatide in SEDDS compared to docusate n-octadecyl sulfate in the study of Thi et al. The molar ratio of n-octadecyl sulfate paired with exenatide is higher than that of docusate paired with exenatide. This means that more n-octadecyl sulfate is required for the same exenatide, which increases the lipophilicity of exenatide and the log P value of exenatide-n-octadecyl sulfate. The ex vivo results showed that the intestinal permeability and bioavailability of n-octadecyl sulfate were 3.5 and 1.3 times higher than those of docusate, respectively [167].

An alternative to hydrophobic ion pairing is to load the peptide into a W/O emulsion, which is then dispersed into SEDDS. Lu et al. used soybean phospholipids to wrap exenatide to form reverse micelles, which were then incorporated into SEDDS. Subsequently, O/W emulsions were prepared on the basis of reverse micelles-SEDDS, the surface of which was positively charged, and finally hyaluronic acid was encapsulated in the outermost layer by electrostatic adsorption. The hyaluronic acid coating adheres to the intestinal mucosa and increases intestinal permeability. Animal experiments showed that exenatide-loaded reverse micelles-SEDDS was more significant in improving the indexes of T2DM rats compared to exenatide-loaded SEDDS. Long-term experiments also showed that long-term administration of exenatide-loaded reverse micelles-SEDDS protects pancreatic β-cells and improves insulin secretion to some extent in T2DM rats [168].

Converting liquid SEDDS to solid SEDDS (S-SEDDS) allows for controlled release of SEDDS. S-SEDDS improves the physicochemical stability and drug loading capacity of SEDDS and offers the advantages of controlled drug release, extended gastric retention time, and reduced production cost. S-SEDDS is prepared by solidifying SEDDS, which is commonly performed by solid carrier adsorption, spray drying, thermosolvent extrusion, and freeze drying [169]. Sun and colleagues prepared cabazitaxel S-SEDDS by solid carrier adsorption, which formed celiac particles into lymphatic transport to avoid the first pass effect, greatly improved the bioavailability of the BCS class IV drug cabazitaxel, and developed more potential formulations of cabazitaxel [170].

### 5.3. Polymer Nanoparticles (PNP)

Nanoparticles prepared from biocompatible and biodegradable polymers are widely used in oral delivery systems. Polymers are classified into natural and synthetic polymers; natural polymers such as polysaccharides, proteins, and polyamino acids have the advantage of being non-toxic and non-immunogenic in oral delivery. Synthetic polymers allow for the flexible production or modification of materials according to patient needs, such as synthetic polymers, silica, porous coordination polymers, etc. [171].

Chitosan is a cationic polysaccharide derived from naturally occurring chitin and therefore adheres to negatively charged mucus layers and cell surfaces. Intestinal epithelial intercellular channels are restricted by a combination of tightly linked protein complexes, cellular bridges, and adherens junctions, whereas chitosan transiently opens tight junctions to increase penetration of the drug into the paracellular pathway, and this process is reversible [172]. Sodium alginate is a PH-sensitive anionic polysaccharide and is adhesive, interacting with the mucus layer through hydrogen bonding and van der Waals forces [173]. Yang and colleagues prepared chitosan sodium alginate gel nanoparticles loaded with exenatide using sodium tripolyphosphate as a cross-linking agent. Due to the PH sensitivity of sodium alginate and the strong electrostatic interaction with chitosan, the nanoparticles shrink under acidic conditions, thereby protecting the exenatide. In the intestinal tract, sodium alginate adheres to the intestinal mucosa, increasing drug retention time, and chitosan increases the permeability of the nanoparticles. In vivo experiments showed that nanoparticles loaded with exenatide had an oral bioavailability of approximately 9% and significantly and consistently reduced blood glucose concentrations in diabetic rats [174].

Protein carriers are abundant and self-targeting, and the structure of the protein itself gives it the potential to deliver RNA, drugs, vaccines, and other biomolecules at specific sites in the body. However, it should be noted that high concentrations of proteins can be denatured by aggregation, and protein solubility can be improved by adding buffer components or low concentrations of surfactants [175]. Bao et al. used zeinolysin to protect the complex formed by the non-covalent interaction of liglutide with bile acids and added a surface-active glycolipid, rhamnolipid, to improve the stability of zeinolysin nanoparticles. Bile acids, as intestinal mucosal pro-osmotic agents, in combination with liraglutide may improve the oral absorption of liraglutide without affecting its potency. Corn Alcohol Soluble Protein, as a hydrophobic protein, can help its encapsulated material to resist protease degradation, while rhamnolipid prevents its hydrophobic aggregation, thereby reducing the particle size of the nanoparticles. Cellular experiments showed that in contrast to the ligraglutide bile acid complex alone, which is absorbed only via the bile acid transporter protein pathway, zeinolysin particles are absorbed via active transport, lattice proteins, vesicles, and microcellular drinking-mediated endocytosis. In vivo experiments revealed that oral administration of corn alcohol-soluble protein loaded with liraglutide had a bioavailability of 9.6% and significantly reduced blood glucose levels in T2DM mice [176].

The most interesting of the current polyamino acids are the amphoteric amino acid polymers, where the average distribution of oppositely charged groups makes them strongly hydrated, and thus the amphoteric amino acid polymers have the advantage of resisting protein adsorption, interfacial lubrication, facilitating mucus permeation, enhancing the internalization of the nanoparticles in the cell, and also preventing degradation of the proteopeptides in inflammatory and enzymatic environments [177]. The degradation products of poly(lactic-co-glycolic) acid, PLGA, are lactic acid and hydroxyacetic acid, which are also by-products of the human metabolic pathway, so when it is in the body, it is not toxic, except for those with lactose deficiency. PLGAs can also be modified in various ways and are therefore often used in the design and development of nanocarriers for controlled and targeted drug delivery systems. Zhao et al. selected amphoteric sulfobetaine for surface modification of PLGA nanoparticles for loading of liraglutide. Amphoteric sulfobetaine can self-assemble on the surface of PLGA nanoparticles to form hydrophilic and electroneutral coatings and to form protein coronas to resist adsorption to nonspecific proteins. The results showed that the hydrophilicity and electroneutrality of the surface of PLGA particles greatly improved their mucus permeability, and the relative bioavailability of the orally administered nanoparticles was 9.95% of that of the subcutaneously administered group, with a stable blood glucose-lowering effect [178].

Silica has the advantages of easy synthesis, large pore size, large specific surface area, good physical stability, non-toxicity, etc. Silica-skeleton-loaded drugs have better stability under temperature changes, organic solvents, or acidic conditions, making them suitable for use as a drug nanocarrier [179]. Silicon dioxide uptake in the GI tract is related to the size of the nanoparticles; e.g., 250 nm particles can be transported via cellular uptake and cytosis, while 100 nm particles result in a lower rate of cellular transport due to faster degradation. Silica particles smaller than 100 nm in size, on the other hand, improved intestinal osmotic penetration, but when penetrating the mucus layer, the penetration rate of 20 nm particles was much lower than that of 50 nm particles [180]. Therefore the relationship between the size of silica nanoparticles and their absorption in the GI tract still needs to be explored. Silica for the preparation of nanoparticles can be classified into mesoporous and non-porous silica, and mesoporous silica can be used for the delivery of peptide drugs. The ordered nanoscale porous structure in mesoporous silica nanoparticles allows drug molecules to be easily confined and stabilized within the mesopore in an amorphous form, which further enhances the drug dissolution rate [181]. Muhammad and colleagues tried macroporous dendritic silica nanoparticles loaded with exenatide, modified the nanoparticles using phosphonate, and covered the surface with a chitosan coating. The phosphonate enhanced the surface charge and porosity of the nanoparticles, resulting in a more homogeneous dispersion and increased exenatide loading. The stronger electronegativity of phosphonate can form electrostatic adsorption with chitosan, and the chitosan-coated nanoparticles can protect the exenatide leakage in the pores and improve the drug loading capacity. In addition, chitosan coating reduced the burst release of exenatide at acidic and alkaline pH, and increased the permeability of nanoparticles in intestinal epithelial cells [182].

Porous coordination polymers are materials with infinite network structures formed by self-assembly of metal nodes (metal ions or clusters) and organic linkers through coordination bonds, which have the advantages of diverse structures and compositions, designable structures, adjustable pore channels, and easy functionalization [183,184,185]. The nanocarriers commonly used in porous coordination polymers for loading drugs are metal-organic frameworks, and functional metal-organic framework nanoparticles can protect proteins from the gastric environment and overcome the intestinal barrier, offering the possibility of orally delivering biomolecules. Jun-Jie Zou et al. Encapsulation of insulin using acid-resistant metal-organic framework nanoparticles and modification of its surface with transferrin for efficient transport and targeted release of insulin [186]. The macroporous structure of metal-organic framework nanoparticles allows for up to 33% encapsulation of insulin and its environmental sensitivity for controlled release under phosphorus-rich physiological conditions. Transferrin achieves iron uptake and transport by binding to the transferrin receptor on epithelial cells, which increases the efficiency of insulin transport. The intracellular nanoparticles, due to their strong positive charge and unique membrane switching effect, interact electrostatically with the lysosomal membrane to achieve rapid escape from the lysosome and release from the cellular monolayer. Animal experiments showed that transferrin-modified metal-organic framework nanoparticles rapidly reduced in vivo blood glucose levels in rats to about 30% of the initial level and extended insulin efficacy up to 10 h, achieving a high oral bioavailability of 29.6%, while long-term experiments demonstrated that such orally-administered nanoparticles are well biocompatible. This demonstrates the potential of metal-organic frameworks for loading peptide drugs for oral treatment of diabetes.

## 6. Conclusions and Outlook

Oral delivery of peptides is one of the major challenges in the field of drug delivery. Currently, the focus of peptide drug formulation research is mainly on protecting the biological activity of peptides and improving bioavailability. Nano-formulations can avoid the inactivation of peptides in the gastrointestinal environment, increase the transmembrane absorption in the small intestinal epithelium, change the distribution of the drug in the body, and improve bioavailability. Therapeutic effects of controlled release and targeted treatment of diseases are achieved by ligand modification, which prolongs retention time at specific absorption sites. In addition, nano-formulations activate cellular transport mechanisms to maximize drug activity, ensuring rapid and stable drug action [187]. This paper contributes to the field of therapeutic diabetes by reviewing the progress of research on peptide drugs for the treatment of diabetes, as well as exploring the possibility of combining peptide drugs with nano-formulations.

## Figures and Tables

**Table 1:** Application of polypeptide drugs in clinical research of type 2 diabetes mellitus.

Type	Molecular Formula	CAS Number	Molecular Weight	Detection Indexes of Type 2 Diabetes Mellitus	Phase	Trial Number	Reference
Exenatide	C184H282N50O60S	141758-74-9	4186.57	Hepatic fat content	Phase 4	NCT01432405	[12]
Lixisenatide	C215H347N61O65S	320367-13-3	4858.49	Blood lipid level	Phase 2	NCT02274740	[13]
Loxenatide	C210H325N55O69S	2420483-82-3	44,212.65	Phase 1	kidney function	NCT02467790	[14]
Liraglutide	C172H265N43O51	204656-20-2	3751.20	Cardiovascular	Phase 4	NCT01761318	[15]
Beinaglutide	C149H225N39O46	123475-27-4	3298.61	Blood sugar	Not Applicable	NCT03829891	[16]
Dulaglutide	C149H221N37O49	923950-08-7	3314.62	Blood pressure, heart rate	Phase 2	NCT01149421	[17]
Semaglutide	C187H291N45O59	910463-68-2	4113.58	Heart disease, cardiovascular	Phase 3	NCT03914326	[18]
Tirzepatide	C225H348N48O68 • XNa	2023788-19-2	4813.45	HbA1c	Phase 4	NCT05706506	[19]

**Table 2:** Comparison of different kinds of oral drugs.

Type	Representative Drug	Function	Advantages	Possible Side Effects	Reference
Polypeptide drugs	Semeglutide	When the blood glucose level rises, it causes the release of insulin It can be used with metformin, basal insulin or sulfonylureas.	It may reduce hunger. May lead to weight loss.	Disgusting. Vomiting. Diarrhoea. Abdominal pain.	[109]
Meglitazone drugs	Reglinet	Triggers pancreas to release insulin	Quick results	Blood sugar levels drop too low, that is, hypoglycemia. Weight gain	[140]
Sulfonylureas	Glipizide	Triggers pancreas to release insulin	low cost. Effectively reduce blood sugar	The blood sugar level dropped too low. Weight gain. Rash. If drinking, there will be nausea or vomiting.	[141]
DPP-4 inhibitors	Saxagliptin	When blood sugar rises, it causes the release of insulin. Limit the ability of the liver to release glucose	Does not lead to weight gain. When used alone or in combination with metformin, it does not cause blood glucose levels to drop too low.	Upper respiratory tract infection. Throat pain. Headache.	[142]
Metformin drug	Metformin	Limit the ability of the liver to release sugar. Improve the sensitivity of cells to insulin	Very effective. May cause a slight weight loss. Low cost.	Nausea. Stomachache. Diarrhoea. In rare cases, there is harmful accumulation of lactic acid in patients with renal or liver failure, that is, lactic acidosis.	[143]
Alpha glucosidase inhibitors	Miglitol	Slow down the body’s ability to decompose starch and some sugars	Does not lead to weight gain. Unless taken with insulin or sulfonylureas, it will not cause blood glucose levels to drop too low.	Bloating Stomachache Diarrhoea	[144]
PPARγ agonists	Pioglitazone	Agonizes peroxisome proliferator-activated receptor PPARγ and reduces insulin resistance	Does not stimulate insulin secretion, but exerts its hypoglycemic effect by enhancing tissue sensitivity to insulin. Used alone, it does not cause hypoglycemia.	Increased risk of heart failure. Increased incidence of bladder cancer	[138]
SGLT-2 inhibitor	Dagliflozin	Inhibits SGLT-2, reduces glucose reabsorption, and excretes sugar directly from the urine.	SGLT-2 inhibitors are rarely hypoglycemic and have beneficial effects on improving body weight and blood pressure	May be associated with nasopharyngitis, fungal infections, and genitourinary infections	[139]

## References

[1] Jin Q., Ma R.C.W. (2021). Metabolomics in Diabetes and Diabetic Complications: Insights from Epidemiological Studies. Cells.

[2] Haak T., Gölz S., Fritsche A., Füchtenbusch M., Siegmund T., Schnellbächer E., Klein H.H., Uebel T., Droßel D. (2019). Therapy of Type 1 Diabetes. Exp. Clin. Endocrinol. Diabetes.

[3] Berbudi A., Rahmadika N., Tjahjadi A.I., Ruslami R. (2020). Type 2 Diabetes and its Impact on the Immune System. Curr. Diabetes Rev..

[4] Pavlou D.I., Paschou S.A., Anagnostis P., Spartalis M., Spartalis E., Vryonidou A., Tentolouris N., Siasos G. (2018). Hypertension in patients with type 2 diabetes mellitus: Targets and management. Maturitas.

[5] Alsaadon H., Afroz A., Karim A., Habib S.H., Alramadan M.J., Billah B., Shetty A.N. (2022). Hypertension and its related factors among patients with type 2 diabetes mellitus—A multi-hospital study in Bangladesh. BMC Public Health.

[6] Wang Z.J., Wang J.Q., Kahkoska A.R., Buse J.B., Gu Z. (2021). Developing Insulin Delivery Devices with Glucose Responsiveness. Trends Pharmacol. Sci..

[7] Goff L.M. (2019). Ethnicity and Type 2 diabetes in the UK. Diabet. Med..

[8] Cloete L. (2021). Diabetes mellitus: An overview of the types, symptoms, complications and management. Nurs. Stand..

[9] Rekha M.R., Sharma C.P. (2013). Oral delivery of therapeutic protein/peptide for diabetes—Future perspectives. Int. J. Pharmaceut..

[10] Bhutani U., Basu T., Majumdar S. (2021). Oral Drug Delivery: Conventional to Long Acting New-Age Designs. Eur. J. Pharm. Biopharm..

[11] Niu Z.G., Conejos-Sánchez I., Griffin B.T., O’Driscoll C.M., Alonso M.J. (2016). Lipid-based nanocarriers for oral peptide delivery. Adv. Drug Deliv. Rev..

[12] Shaw J.E., Gallwitz B., Han J., Hardy E., Schernthaner G. (2017). Variability in and predictors of glycaemic responses after 24 weeks of treatment with exenatide twice daily and exenatide once weekly. Diabetes Obes. Metab..

[13] Denimal D., Bergas V., Pais-de-Barros J.-P., Simoneau I., Demizieux L., Passilly-Degrace P., Bouillet B., Petit J.-M., Rouland A., Bataille A. (2023). Liraglutide reduces plasma dihydroceramide levels in patients with type 2 diabetes. Cardiovasc. Diabetol..

[14] Gao F., Lv X., Mo Z., Ma J., Zhang Q., Yang G., Liu W., Li Q., Zhou J., Bao Y. (2020). Efficacy and safety of polyethylene glycol loxenatide as add-on to metformin in patients with type 2 diabetes: A multicentre, randomized, double-blind, placebo-controlled, phase 3b trial. Diabetes Obes. Metab..

[15] Tack C., Jacob S., Desouza C., Bain S.C., Nauck M.A., Petrie J., Poulter N.R., Pratley R.E., Stegmann H.V.B.K., Bosch-Traberg H. (2018). Liraglutide Effects in Insulin-Treated Patients in LEADER. Diabetes.

[16] Xiangyang L., Wenjuan Y., Jianrong L., Xinxi H., Yujie F., Jie M., Jingbo L., Jianfang F., Qiuhe J., Li W. (2023). The efficacy and safety of beinaglutide alone or in combination with insulin glargine in Chinese patients with type 2 diabetes mellitus who are inadequately controlled with oral antihyperglycemic therapy: A multicenter, open-label, randomized trial. J. Diabetes.

[17] Bowe A., Poonawalla I., Tindal M., Meah Y.A., Schwab P. (2020). 1428-P: Cardiovascular Outcomes with Dulaglutide vs. Liraglutide. Diabetes.

[18] McGuire D.K., Busui R.P., Deanfield J., Inzucchi S.E., Mann J.F.E., Marx N., Mulvagh S.L., Poulter N., Engelmann M.D.M., Hovingh G.K. (2023). Effects of oral semaglutide on cardiovascular outcomes in individuals with type 2 diabetes and established atherosclerotic cardiovascular disease and/or chronic kidney disease: Design and baseline characteristics of SOUL, a randomized trial. Diabetes Obes. Metab..

[19] Karagiannis T., Avgerinos I., Liakos A., Del Prato S., Matthews D.R., Tsapas A., Bekiari E. (2022). Management of type 2 diabetes with the dual GIP/GLP-1 receptor agonist tirzepatide: A systematic review and meta-analysis. Diabetologia.

[20] Drucker D.J. (2018). Mechanisms of Action and Therapeutic Application of Glucagon-like Peptide-1. Cell Metab..

[21] Müller T.D., Finan B., Bloom S.R., D’Alessio D., Drucker D.J., Flatt P.R., Fritsche A., Gribble F., Grill H.J., Habener J.F. (2019). Glucagon-like peptide 1 (GLP-1). Mol. Metab..

[22] Tucker J.D., Dhanvantari S., Brubaker P.L. (1996). Proglucagon processing in islet and intestinal cell lines. Regul. Pept..

[23] Reimann F., Gribble F.M. (2016). Mechanisms underlying glucose-dependent insulinotropic polypeptide and glucagon-like peptide-1 secretion. J. Diabetes Investig..

[24] Ezcurra M., Reimann F., Gribble F.M., Emery E. (2013). Molecular mechanisms of incretin hormone secretion. Curr. Opin. Pharmacol..

[25] Ahrén B. (2011). GLP-1 for type 2 diabetes. Exp. Cell Res..

[26] Nauck M.A., Quast D.R., Wefers J., Meier J.J. (2020). GLP-1 receptor agonists in the treatment of type 2 diabetes—State-of-the-art. Mol. Metab..

[27] Vasu S., Moffett R.C., Thorens B., Flatt P.R. (2014). Role of endogenous GLP-1 and GIP in beta cell compensatory responses to insulin resistance and cellular stress. PLoS ONE.

[28] De Marinis Y.Z., Salehi A., Ward C.E., Zhang Q., Abdulkader F., Bengtsson M., Braha O., Braun M., Ramracheya R., Amisten S. (2010). GLP-1 inhibits and adrenaline stimulates glucagon release by differential modulation of N- and L-type Ca2+ channel-dependent exocytosis. Cell Metab..

[29] Drucker D.J., Habener J.F., Holst J.J. (2017). Discovery, characterization, and clinical development of the glucagon-like peptides. J. Clin. Investig..

[30] Baggio L.L., Yusta B., Mulvihill E.E., Cao X., Streutker C.J., Butany J., Cappola T.P., Margulies K.B., Drucker D.J. (2018). GLP-1 Receptor Expression Within the Human Heart. Endocrinology.

[31] Sonne D.P., Engstrøm T., Treiman M. (2007). Protective effects of GLP-1 analogues exendin-4 and GLP-1(9-36) amide against ischemia-reperfusion injury in rat heart. Regul. Pept..

[32] Helmstädter J., Keppeler K., Küster L., Münzel T., Daiber A., Steven S. (2021). Glucagon-like peptide-1 (GLP-1) receptor agonists and their cardiovascular benefits—The role of the GLP-1 receptor. Br. J. Pharmacol..

[33] Smith N.K., Hackett T.A., Galli A., Flynn C.R. (2019). GLP-1: Molecular mechanisms and outcomes of a complex signaling system. Neurochem. Int..

[34] Holst J.J. (2007). The physiology of glucagon-like peptide 1. Physiol. Rev..

[35] Trapp S., Brierley D.I. (2021). Brain GLP-1 and the regulation of food intake: GLP-1 action in the brain and its implications for GLP-1 receptor agonists in obesity treatment. Br. J. Pharmacol..

[36] Xu F., Lin B., Zheng X., Chen Z., Cao H., Xu H., Liang H., Weng J. (2016). GLP-1 receptor agonist promotes brown remodelling in mouse white adipose tissue through SIRT1. Diabetologia.

[37] Giralt M., Vergara P. (1998). Sympathetic pathways mediate GLP-1 actions in the gastrointestinal tract of the rat. Regul. Pept..

[38] Jiménez D.L., Babkowski M.C., Miramontes González J.P. (2019). GLP-1 and the renin-angiotensin-aldosterone system. Lancet Diabetes Endocrinol..

[39] Han L., Chen X., Wan D., Xie M., Ouyang S. (2024). One anastomosis gastric bypass ameliorates diabetic nephropathy via regulating the GLP-1-mediated Sirt1/AMPK/PGC1α pathway. Clin. Exp. Nephrol..

[40] Niu B., Fang H., Chen Q. (2023). The Discovery and Development of Glucagon-Like Peptide-1 Receptor Agonists. Curr. Med. Chem..

[41] Davidson M.B., Bate G., Kirkpatrick P. (2005). Exenatide. Nat. Rev. Drug Discov..

[42] Aart-van der Beek A.B., Raalte D.H., Guja C., Hoogenberg K., Suchower L.J., Hardy E., Sjöström C.D., Heerspink H.J.L. (2020). Exenatide once weekly decreases urinary albumin excretion in patients with type 2 diabetes and elevated albuminuria: Pooled analysis of randomized active controlled clinical trials. Diabetes Obes. Metab..

[43] Wang M.-J., Cai X., Liang R.-Y., Zhang E.-M., Liang X.-Q., Liang H., Fu C., Zhou A.-D., Shi Y., Xu F. (2023). SIRT1-dependent deacetylation of Txnip H3K9ac is critical for exenatide-improved diabetic kidney disease. Biomed. Pharmacother..

[44] Tsapas A., Karagiannis T., Kakotrichi P., Avgerinos I., Mantsiou C., Tousinas G., Manolopoulos A., Liakos A., Malandris K., Matthews D.R. (2021). Comparative efficacy of glucose-lowering medications on body weight and blood pressure in patients with type 2 diabetes: A systematic review and network meta-analysis. Diabetes Obes. Metab..

[45] Drucker D.J., Buse J.B., Taylor K., Kendall D.M., Trautmann M., Zhuang D., Porter L. (2008). Exenatide once weekly versus twice daily for the treatment of type 2 diabetes: A randomised, open-label, non-inferiority study. Lancet.

[46] Lv Q., Yang Y., Lv Y., Wu Q., Hou X., Li L., Ye X., Yang C., Wang S. (2024). Long-term effects of different hypoglycemic drugs on carotid intima-media thickness progression: A systematic review and network meta-analysis. Front. Endocrinol..

[47] Zhou Q., Hao G., Xie W., Chen B., Lu W., Wang G., Zhong R., Chen J., Ye J., Shen J. (2024). Exenatide reduces atrial fibrillation susceptibility by inhibiting hKv1.5 and hNav1.5 channels. J. Biol. Chem..

[48] DeYoung M.B., MacConell L., Sarin V., Trautmann M., Herbert P. (2011). Encapsulation of exenatide in poly-(D,L-lactide-co-glycolide) microspheres produced an investigational long-acting once-weekly formulation for type 2 diabetes. Diabetes Technol. Ther..

[49] Varanko A.K., Chilkoti A. (2019). Molecular and Materials Engineering for Delivery of Peptide Drugs to Treat Type 2 Diabetes. Adv. Healthc. Mater..

[50] Cai H., Chen Q., Duan Y., Zhao Y., Zhang X. (2023). Short-term effect of polyethylene glycol loxenatide on weight loss in overweight or obese patients with type 2 diabetes: An open-label, parallel-arm, randomized, metformin-controlled trial. Front. Endocrinol..

[51] Wu Y., Guo Z., Wang J., Wang Y., Wang D., Li Y., Zhu L., Sun X. (2021). Polyethylene Glycol Loxenatide (PEX-168) Reduces Body Weight and Blood Glucose in Simple Obese Mice. Int. J. Endocrinol..

[52] Chen F., He L., Li J., Yang S., Zhang B., Zhu D., Wu Z., Zhang S., Hou D., Ouyang C. (2022). Polyethylene Glycol Loxenatide Injection (GLP-1) Protects Vascular Endothelial Cell Function in Middle-Aged and Elderly Patients with Type 2 Diabetes by Regulating Gut Microbiota. Front. Mol. Biosci..

[53] Xie Y., Kuang J., Li Q., Hong T., Ji L., Kong Y., Duan Y., Chen L. (2023). Impact of polyethylene glycol loxenatide on cardiovascular outcomes in patients with type 2 diabetes: Study protocol for a multicentre, randomised, double-blind, placebo-controlled trial (BALANCE-3). BMJ Open.

[54] Yuan J., Wang Y., Wang D., Yan H., Wang N. (2023). Loxenatide attenuates ROS-mediated vascular endothelial progenitor cell damage and mitochondrial dysfunction via SIRT3/Foxo3 signaling pathway. J. Biochem. Mol. Toxicol..

[55] Thorkildsen C., Neve S., Larsen B.D., Meier E., Petersen J.S. (2003). Glucagon-like peptide 1 receptor agonist ZP10A increases insulin mRNA expression and prevents diabetic progression in db/db mice. J. Pharmacol. Exp. Ther..

[56] Raccah D., Gourdy P., Sagnard L., Ceriello A. (2014). Lixisenatide as add-on to oral anti-diabetic therapy: An effective treatment for glycaemic control with body weight benefits in type 2 diabetes. Diabetes Metab. Res..

[57] Trujillo J.M., Roberts M., Dex T., Chao J., White J., LaSalle J. (2018). Low Incidence of Gastrointestinal Adverse Events Over Time with a Fixed-Ratio Combination of Insulin Glargine and Lixisenatide vs Lixisenatide Alone. Diabetes Obes. Metab..

[58] Scott L.J. (2013). Lixisenatide: A review of its use in patients with type 2 diabetes mellitus. BioDrugs.

[59] Scott L.J. (2017). Insulin Glargine/Lixisenatide: A Review in Type 2 Diabetes. Drugs.

[60] Rayner C.K., Watson L.E., Phillips L.K., Lange K., Bound M.J., Grivell J., Wu T., Jones K.L., Horowitz M., Ferrannini E. (2020). Effects of Sustained Treatment with Lixisenatide on Gastric Emptying and Postprandial Glucose Metabolism in Type 2 Diabetes: A Randomized Controlled Trial. Diabetes Care.

[61] Vinué Á., Navarro J., Herrero-Cervera A., García-Cubas M., Andrés-Blasco I., Martínez-Hervás S., Real J.T., Ascaso J.F., González-Navarro H. (2017). The GLP-1 analogue lixisenatide decreases atherosclerosis in insulin-resistant mice by modulating macrophage phenotype. Diabetologia.

[62] Xu L., Chen G., Zhang L., He A., Li Y. (2023). Lixisenatide ameliorated lipopolysaccharide (LPS)-induced expression of mucin and inflammation in bronchial epithelial cells. J. Biochem. Mol. Toxicol..

[63] Zhao Z., Pu Y. (2019). Lixisenatide enhances mitochondrial biogenesis and function through regulating the CREB/PGC-1α pathway. Biochem. Biophys. Res. Commun..

[64] Christensen M., Knop F.K., Vilsbøll T., Holst J.J. (2011). Lixisenatide for type 2 diabetes mellitus. Expert Opin. Investig. Drugs.

[65] Hanefeld M., Arteaga J.M., Leiter L.A., Marchesini G., Nikonova E., Shestakova M., Stager W., Gómez-Huelgas R. (2017). Efficacy and safety of lixisenatide in patients with type 2 diabetes and renal impairment. Diabetes Obes. Metab..

[66] Lin C.-H., Shao L., Zhang Y.-M., Tu Y.-J., Zhang Y., Tomlinson B., Chan P., Liu Z. (2019). An evaluation of liraglutide including its efficacy and safety for the treatment of obesity. Expert Opin. Pharmacother..

[67] Drucker D.J., Dritselis A., Kirkpatrick P. (2010). Liraglutide. Nat. Rev. Drug Discov..

[68] Vandemark C., Nguyen J., Zhao Z.-Q. (2023). Cardiovascular Protection with a Long-Acting GLP-1 Receptor Agonist Liraglutide: An Experimental Update. Molecules.

[69] Qiao H., Ren H., Du H., Zhang M., Xiong X., Lv R. (2018). Liraglutide repairs the infarcted heart: The role of the SIRT1/Parkin/mitophagy pathway. Mol. Med. Rep..

[70] Le Y., Wei R., Yang K., Lang S., Gu L., Liu J., Hong T., Yang J. (2020). Liraglutide ameliorates palmitate-induced oxidative injury in islet microvascular endothelial cells through GLP-1 receptor/PKA and GTPCH1/eNOS signaling pathways. Peptides.

[71] Mashayekhi M., Beckman J.A., Nian H., Garner E.M., Mayfield D., Devin J.K., Koethe J.R., Brown J.D., Cahill K.N., Yu C. (2022). Comparative effects of weight loss and incretin-based therapies on vascular endothelial function, fibrinolysis and inflammation in individuals with obesity and prediabetes: A randomized controlled trial. Diabetes Obes. Metab..

[72] Liang R., Wang M., Fu C., Liang H., Deng H., Tan Y., Xu F., Cai M. (2020). Liraglutide protects against high-fat diet-induced kidney injury by ameliorating apoptosis. Endocr. Connect..

[73] Slomski A. (2017). Liraglutide May Reduce Diabetic Kidney Disease. JAMA.

[74] Wronka M., Krzemińska J., Młynarska E., Rysz J., Franczyk B. (2023). New Insights into the Use of Liraglutide—Impact on Cardiovascular Risk and Microvascular Outcomes. Biomedicines.

[75] Mann J.F.E., Ørsted D.D., Brown-Frandsen K., Marso S.P., Poulter N.R., Rasmussen S., Tornøe K., Zinman B., Buse J.B. (2017). Liraglutide and Renal Outcomes in Type 2 Diabetes. N. Engl. J. Med..

[76] Li J., Li N., Yan S., Lu Y., Miao X., Gu Z., Shao Y. (2019). Liraglutide protects renal mesangial cells against hyperglycemia-mediated mitochondrial apoptosis by activating the ERK-Yap signaling pathway and upregulating Sirt3 expression. Mol. Med. Rep..

[77] Liang R., Wang M., Fu C., Xu F.E.N., Cai M. (2019). 2032-P: The Effects of Liraglutide on Ameliorating Diabetic Kidney Disease Is via SIRT1/TXNIP Pathway. Diabetes.

[78] Fang X., Du Z., Duan C., Zhan S., Wang T., Zhu M., Shi J., Meng J., Zhang X., Yang M. (2021). Beinaglutide shows significantly beneficial effects in diabetes/obesity-induced nonalcoholic steatohepatitis in ob/ob mouse model. Life Sci..

[79] Zhang Y.L., Zhou C., Li X.F., Yang M.N., Tao L., Zheng X.Y., Jia Y.S. (2019). Beinaglutide showed significant weight-loss benefit and effective glycaemic control for the treatment of type 2 diabetes in a real-world setting: A 3-month, multicentre, observational, retrospective, open-label study. Obes. Sci. Pract..

[80] Gao L., Huang H., Zhang L., Zhang N., Fu Y., Zhu D., Bi Y., Feng W. (2021). Comparison of Beinaglutide Versus Metformin for Weight Loss in Overweight and Obese Non-diabetic Patients. Exp. Clin. Endocrinol. Diabetes.

[81] Wen Q., Fang S., Liang Y., Tian Y., Chen Y., Yuan J., Chen Q. (2023). Short-term effect of beinaglutide combined with metformin versus metformin alone on weight loss and metabolic profiles in obese patients with polycystic ovary syndrome: A pilot randomized trial. Front. Endocrinol..

[82] Wang G.-Y., Wang H.-Q., Zhang F.-J., Jiao A.-F., Li Y.-Y., Zhang J.-M., Huang Z.-L., Gao Y.-H., Chi Y.-J., Ma C.-M. (2020). The effect of beinaglutide on visceral fat and bodyweight in obese type 2 diabetic patients. Arch. Med. Sci..

[83] Zhang F., Chen Z., Wu D., Tian L., Chen Q., Ye Y., Chen W., Wu X., Wu P., Yuan W. (2021). Recombinant human GLP-1 beinaglutide regulates lipid metabolism of adipose tissues in diet-induced obese mice. iScience.

[84] Wang G., Wu P., Qiu Y., Dong X., Wang Y., Chi Y., Zhang F., Li Y., Zhang J., Huang Z. (2021). Effect of beinaglutide treatment on weight loss in Chinese patients with type 2 diabetes mellitus and overweight/obesity. Arch. Endocrinol. Metab..

[85] Sanford M. (2014). Dulaglutide: First global approval. Drugs.

[86] Jendle J., Grunberger G., Blevins T., Giorgino F., Hietpas R.T., Botros F.T. (2016). Efficacy and safety of dulaglutide in the treatment of type 2 diabetes: A comprehensive review of the dulaglutide clinical data focusing on the AWARD phase 3 clinical trial program. Diabetes Metab. Res. Rev..

[87] Scott L.J. (2020). Dulaglutide: A Review in Type 2 Diabetes. Drugs.

[88] Frias J.P., Bonora E., Nevarez Ruiz L., Li Y.G., Yu Z., Milicevic Z., Malik R., Bethel M.A., Cox D.A. (2021). Efficacy and Safety of Dulaglutide 3.0 mg and 4.5 mg Versus Dulaglutide 1.5 mg in Metformin-Treated Patients with Type 2 Diabetes in a Randomized Controlled Trial (AWARD-11). Diabetes Care.

[89] Kimura T., Obata A., Shimoda M., Hirukawa H., Kanda-Kimura Y., Nogami Y., Kohara K., Nakanishi S., Mune T., Kaku K. (2018). Durability of protective effect of dulaglutide on pancreatic β-cells in diabetic mice: GLP-1 receptor expression is not reduced despite long-term dulaglutide exposure. Diabetes Metab..

[90] Zheng W., Pan H., Wei L., Gao F., Lin X. (2019). Dulaglutide mitigates inflammatory response in fibroblast-like synoviocytes. Int. Immunopharmacol..

[91] Yifan W., Yixiang W., Yang W., Yiqing W. (2023). Dulaglutide Ameliorates Intrauterine Adhesion by Suppressing Inflammation and Epithelial–Mesenchymal Transition Via Inhibiting the TGF-β/Smad2 Signaling Pathway. Pharmaceuticals.

[92] Wang Y., Deng F., Zhong X., Du Y., Fan X., Su H., Pan T. (2023). Dulaglutide provides protection against sepsis-induced lung injury in mice by inhibiting inflammation and apoptosis. Eur. J. Pharmacol..

[93] Ferdinand K.C., Dunn J., Nicolay C., Sam F., Blue E.K., Wang H. (2023). Weight-dependent and weight-independent effects of dulaglutide on blood pressure in patients with type 2 diabetes. Cardiovasc. Diabetol..

[94] Konig M., Riddle M.C., Colhoun H.M., Branch K.R., Atisso C.M., Lakshmanan M.C., Mody R., Raha S., Gerstein H.C. (2021). Exploring potential mediators of the cardiovascular benefit of dulaglutide in type 2 diabetes patients in REWIND. Cardiovasc. Diabetol..

[95] Hertzel C.G., Shun-Fu L., Guillaume P., Bethel M.A., Helen M.C., Anastasia H., Mark L., Yanzhu L., Valentino P., Hui-Rong Q. (2023). Biomarker Changes Associated with Both Dulaglutide and Cardiovascular Events in the REWIND Randomized Controlled Trial: A Nested Case-Control Post Hoc Analysis. Diabetes Care.

[96] Gerstein H.C., Colhoun H.M., Dagenais G.R., Diaz R., Lakshmanan M., Pais P., Probstfield J., Riesmeyer J.S., Riddle M.C., Rydén L. (2019). Dulaglutide and cardiovascular outcomes in type 2 diabetes (REWIND): A double-blind, randomised placebo-controlled trial. Lancet.

[97] Luo X., Hu Y., He S., Ye Q., Lv Z., Liu J., Chen X. (2019). Dulaglutide inhibits high glucose- induced endothelial dysfunction and NLRP3 inflammasome activation. Arch. Biochem. Biophys..

[98] Mei X., Li Y., Wu J., Liao L., Lu D., Qiu P., Yang H.-l., Tang M.-w., Liang X.-y., Liu D. (2024). Dulaglutide restores endothelial progenitor cell levels in diabetic mice and mitigates high glucose-induced endothelial injury through SIRT1-mediated mitochondrial fission. Biochem. Biophys. Res. Commun..

[99] Dhillon S. (2018). Semaglutide: First Global Approval. Drugs.

[100] Bucheit J.D., Pamulapati L.G., Carter N., Malloy K., Dixon D.L., Sisson E.M. (2019). Oral Semaglutide: A Review of the First Oral Glucagon-Like Peptide 1 Receptor Agonist. Diabetes Technol. Ther..

[101] Hedrington M.S., Davis S.N. (2018). Oral semaglutide for the treatment of type 2 diabetes. Expert Opin. Pharmacother..

[102] Anderson S.L., Beutel T.R., Trujillo J.M. (2020). Oral semaglutide in type 2 diabetes. J. Diabetes Its Complicat..

[103] Baekdal T.A., Donsmark M., Hartoft-Nielsen M.-L., Søndergaard F.L., Connor A. (2021). Relationship Between Oral Semaglutide Tablet Erosion and Pharmacokinetics: A Pharmacoscintigraphic Study. Clin. Pharmacol. Drug Dev..

[104] Bækdal T.A., Breitschaft A., Donsmark M., Maarbjerg S.J., Søndergaard F.L., Borregaard J. (2021). Effect of Various Dosing Conditions on the Pharmacokinetics of Oral Semaglutide, a Human Glucagon-Like Peptide-1 Analogue in a Tablet Formulation. Diabetes Ther..

[105] Gallwitz B., Giorgino F. (2021). Clinical Perspectives on the Use of Subcutaneous and Oral Formulations of Semaglutide. Front. Endocrinol..

[106] Li A., Su X., Hu S., Wang Y. (2023). Efficacy and safety of oral semaglutide in type 2 diabetes mellitus: A systematic review and meta-analysis. Diabetes Res. Clin. Pract..

[107] Davies M., Pieber T.R., Hartoft-Nielsen M.-L., Hansen O.K.H., Jabbour S., Rosenstock J. (2017). Effect of Oral Semaglutide Compared with Placebo and Subcutaneous Semaglutide on Glycemic Control in Patients with Type 2 Diabetes. JAMA.

[108] Smits M.M., Van Raalte D.H. (2021). Safety of Semaglutide. Front. Endocrinol..

[109] James A., Riddhi P.D., Joshua N., Xi T.A.N., Lin X.I.E., Cory L.G., Mico G., Justin C., Aaron A.K. (2023). 86-LB: Real-World Impact of Oral Semaglutide on Glycemic Control and Weight Outcomes in Type 2 Diabetes (RELATE–Oral Semaglutide). Diabetes.

[110] James A., Riddhi P.D., Joshua N., Xi T.A.N., Lin X.I.E., Cory L.G., Mico G., Justin C., Aaron A.K. (2023). 87-LB: Real-World Impact of Once-Weekly Injectable Semaglutide on Glycemic Control and Weight Outcomes in Type 2 Diabetes (RELATE-OW Injectable Semaglutide). Diabetes.

[111] Aroda V.R., Erhan U., Jelnes P., Meier J.J., Abildlund M.T., Pratley R., Vilsbøll T., Husain M. (2023). Safety and tolerability of semaglutide across the SUSTAIN and PIONEER phase IIIa clinical trial programmes. Diabetes Obes. Metab..

[112] Priya M., Arya M.S. (2023). Oral semaglutide: An OASIS from injectables. Lancet.

[113] Selvarajan R., Subramanian R. (2023). A Peptide in a Pill—Oral Semaglutide in the Management of Type 2 Diabetes. Diabetes Metab. Syndr. Obes. Targets Ther..

[114] Irene F.-R. (2023). Semaglutide is beneficial in patients with HFpEF and obesity. Nat. Rev. Cardiol..

[115] Javed B., Steen Z.A., Barry A.B., Melanie J.D., Dalane W.K., Mark C.P., Sanjiv J.S., Subodh V., Walter P.A., Vijay C. (2023). Semaglutide Effects According to Ejection Fraction in Heart Failure with Preserved Ejection Fraction and Obesity. J. Am. Coll. Cardiol..

[116] Li X., Luo W., Tang Y., Wu J., Zhang J., Chen S., Zhou L., Tao Y., Tang Y., Wang F. (2024). Semaglutide attenuates doxorubicin-induced cardiotoxicity by ameliorating BNIP3-Mediated mitochondrial dysfunction. Redox Biol..

[117] Yan M., Lin K., Huang D., Li J., Qu X., Chen K. (2024). Semaglutide attenuates pathological electrophysiological remodeling in diabetic cardiomyopathy via restoring Cx43 expression. Endocrine.

[118] Coenraad W., Laura M.G.M., Edgar E.N., Schouten E.M., Marie A.S., Lotte B.K., Kristoffer N., Christian T.M., Annabelle H., Gianluca M. (2023). The Cardioprotective Effects of Semaglutide Exceed Those of Dietary Weight Loss in Mice with HFpEF. JACC Basic Transl. Sci..

[119] Mikhail N.K., Steen Z.A., Barry A.B., Javed B., Søren R., Melanie D., Hovingh G.K., Dalane W.K., Marie L.L., Daniél V.M. (2023). Semaglutide in Patients with Heart Failure with Preserved Ejection Fraction and Obesity. N. Engl. J. Med..

[120] Maria Marques V., Paula L., Paula Sanchez B., María Victoria López I., Jose M.P. (2023). #6005 Comparison of Semaglutide Oral vs Subcutaneous in Chronic Kidney Disease (CKD). Nephrol. Dial. Transplant..

[121] Chen X., Chen S., Ren Q., Niu S., Pan X., Yue L., Li Z., Zhu R., Jia Z., Chen X. (2022). Metabolomics Provides Insights into Renoprotective Effects of Semaglutide in Obese Mice. Drug Des. Dev. Ther..

[122] Michael C., Louise S.D., Martin Rønn M., Henrik Björk H., Rune I., Mette Viberg Ø. (2023). #5826 Nephroprotective Effects of Semaglutide in a Mouse Model of Hypertension-Accelerated Diabetic Kidney Disease. Nephrol. Dial. Transplant..

[123] Syed Y.Y. (2022). Tirzepatide: First Approval. Drugs.

[124] Gasbjerg L.S., Bergmann N.C., Stensen S., Christensen M.B., Rosenkilde M.M., Holst J.J., Nauck M., Knop F.K. (2019). Evaluation of the incretin effect in humans using GIP and GLP-1 receptor antagonists. Peptides.

[125] Rhodes R.S.S., Singh S.K., Rajendran V.M., Walk S.T., Coon S.D. (2022). Regulation of Glucose Insulinotropic Peptide and Intestinal Glucose Transporters in the Diet-Induced Obese Mouse. J. Diabetes Res..

[126] Vilsbøll T., Knop F.K., Krarup T., Johansen A., Madsbad S., Larsen S., Hansen T., Pedersen O., Holst J.J. (2003). The pathophysiology of diabetes involves a defective amplification of the late-phase insulin response to glucose by glucose-dependent insulinotropic polypeptide-regardless of etiology and phenotype. J. Clin. Endocrinol. Metab..

[127] Baggio L.L., Drucker D.J. (2020). Glucagon-like Peptide-1 Receptor co-agonists for the treatment of metabolic disease. Mol. Metab..

[128] Holst J.J., Rosenkilde M.M. (2020). GIP as a Therapeutic Target in Diabetes and Obesity: Insight from Incretin Co-agonists. J. Clin. Endocrinol. Metab..

[129] Thomas M.K., Nikooienejad A., Bray R., Cui X., Wilson J., Duffin K., Milicevic Z., Haupt A., Robins D.A. (2020). Dual GIP and GLP-1 Receptor Agonist Tirzepatide Improves Beta-cell Function and Insulin Sensitivity in Type 2 Diabetes. J. Clin. Endocrinol. Metab..

[130] Coskun T., Sloop K.W., Loghin C., Alsina-Fernandez J., Urva S., Bokvist K.B., Cui X., Briere D.A., Cabrera O., Roell W.C. (2018). LY3298176, a novel dual GIP and GLP-1 receptor agonist for the treatment of type 2 diabetes mellitus: From discovery to clinical proof of concept. Mol. Metab..

[131] Heise T., Mari A., DeVries J.H., Urva S., Li J., Pratt E.J., Coskun T., Thomas M.K., Mather K.J., Haupt A. (2022). Effects of subcutaneous tirzepatide versus placebo or semaglutide on pancreatic islet function and insulin sensitivity in adults with type 2 diabetes: A multicentre, randomised, double-blind, parallel-arm, phase 1 clinical trial. Lancet Diabetes Endocrinol..

[132] El K., Douros J.D., Willard F.S., Novikoff A., Sargsyan A., Perez-Tilve D., Wainscott D.B., Yang B., Chen A., Wothe D. (2023). The incretin co-agonist tirzepatide requires GIPR for hormone secretion from human islets. Nat. Metab..

[133] Garvey W.T., Frias J.P., Jastreboff A.M., le Roux C.W., Sattar N., Aizenberg D., Mao H., Zhang S., Ahmad N.N., Bunck M.C. (2023). Tirzepatide once weekly for the treatment of obesity in people with type 2 diabetes (SURMOUNT-2): A double-blind, randomised, multicentre, placebo-controlled, phase 3 trial. Lancet.

[134] NamKoong C., Kim M.S., Jang B.-T., Lee Y.H., Cho Y.-M., Choi H.J. (2017). Central administration of GLP-1 and GIP decreases feeding in mice. Biochem. Biophys. Res. Commun..

[135] Zhang Q., Delessa C.T., Augustin R., Bakhti M., Colldén G., Drucker D.J., Feuchtinger A., Caceres C.G., Grandl G., Harger A. (2021). The glucose-dependent insulinotropic polypeptide (GIP) regulates body weight and food intake via CNS-GIPR signaling. Cell Metab..

[136] Heerspink H.J.L., Sattar N., Pavo I., Haupt A., Duffin K.L., Yang Z., Wiese R.J., Tuttle K.R., Cherney D.Z.I. (2022). Effects of tirzepatide versus insulin glargine on kidney outcomes in type 2 diabetes in the SURPASS-4 trial: Post-hoc analysis of an open-label, randomised, phase 3 trial. Lancet Diabetes Endocrinol..

[137] Wilson J.M., Lin Y., Considine G., Cox A.L., Bowsman L.M., Robins D.A., Riesmeyer J.S., Haupt A., Duffin K., Ruotolo G. (2020). Abstract 13426: The Dual GIP/GLP-1 Receptor Agonist Tirzepatide Improves Cardiovascular Risk Protein Biomarkers in Patients with Type 2 Diabetes. Circulation.

[138] Lian J., Fu J. (2021). Pioglitazone for NAFLD Patients with Prediabetes or Type 2 Diabetes Mellitus: A Meta-analysis. Front. Endocrinol..

[139] Lukic N., Macvanin M.T., Gluvic Z., Rizzo M., Radak D., Suri J.S., Isenovic E.R. (2023). SGLT-2 Inhibitors: The Next-generation Treatment for Type 2 Diabetes Mellitus. Curr. Med. Chem..

[140] Repaglinide S.L.J. (2012). Repaglinide: A review of its use in type 2 diabetes mellitus (vol 72, pg 249, 2012). Drugs.

[141] Tomlinson B., Patil N.G., Fok M., Chan P., Lam C.W.K. (2022). The role of sulfonylureas in the treatment of type 2 diabetes. Expert Opin. Pharmaco..

[142] Suenaga A., Sawa N., Oba Y., Ikuma D., Sekine A., Hasegawa E., Mizuno H., Suwabe T., Ikeda S., Tsujimoto T. (2023). Dipeptidyl peptidase-4 inhibitor-related renal disease. J. Diabetes Complicat..

[143] Sanchez-Rangel E., Inzucchi S.E. (2017). Metformin: Clinical use in type 2 diabetes. Diabetologia.

[144] Mushtaq A., Azam U., Mehreen S., Naseer M.M. (2023). Synthetic α-glucosidase inhibitors as promising anti-diabetic agents: Recent developments and future challenges. Eur. J. Med. Chem..

[145] Kawai T., Sun B., Yoshino H., Feng D., Suzuki Y., Fukazawa M., Nagao S., Wainscott D.B., Showalter A.D., Droz B.A. (2020). Structural basis for GLP-1 receptor activation by LY3502970, an orally active nonpeptide agonist [Pharmacology]. Proc. Natl. Acad. Sci. USA.

[146] Frias J.P., Hsia S., Eyde S., Liu R., Ma X., Konig M., Kazda C., Mather K.J., Haupt A., Pratt E. (2023). Efficacy and safety of oral orforglipron in patients with type 2 diabetes: A multicentre, randomised, dose-response, phase 2 study. Lancet.

[147] Ono R., Furihata K., Ichikawa Y., Nakazuru Y., Bergman A., Gorman D.N., Saxena A.R. (2022). 339-OR: Oral Small-Molecule GLP-1R Agonist Danuglipron Robustly Reduces Plasma Glucose and Body Weight after Eight Weeks in Japanese Adults with Type 2 Diabetes Mellitus. Diabetes.

[148] Saxena A.R., Gorman D.N., Esquejo R.M., Bergman A., Chidsey K., Buckeridge C., Griffith D.A., Kim A.M. (2021). Danuglipron (PF-06882961) in type 2 diabetes: A randomized, placebo-controlled, multiple ascending-dose phase 1 trial. Nat. Med..

[149] Saxena A.R., Frias J.P., Brown L.S., Gorman D.N., Vasas S., Tsamandouras N., Birnbaum M.J. (2023). Efficacy and Safety of Oral Small Molecule Glucagon-Like Peptide 1 Receptor Agonist Danuglipron for Glycemic Control Among Patients with Type 2 Diabetes: A Randomized Clinical Trial. JAMA Netw. Open.

[150] Wu J., Zhou R., Zhang Q., Zhang Q., Qin H., Ye Z., Xu Y., Feng S., Shu C., Shen Y. (2023). Safety, pharmacokinetics and pharmacodynamics of HRS-7535, a novel oral small molecule glucagon-like peptide-1 receptor agonist, in healthy participants: A phase 1, randomized, double-blind, placebo-controlled, single- and multiple-ascending dose, and food effect trial. Diabetes Obes. Metab..

[151] Ma T., Lu W., Wang Y., Qian P., Tian H., Gao X., Yao W. (2021). An oral GLP-1 and GIP dual receptor agonist improves metabolic disorders in high fat-fed mice. Eur. J. Pharmacol..

[152] He H., Lu Y., Qi J., Zhu Q., Chen Z., Wu W. (2019). Adapting liposomes for oral drug delivery. Acta Pharm. Sin. B.

[153] He Y., Huang Y., Xu H., Yang X., Liu N., Xu Y., Ma R., Zhai J., Ma Y., Guan S. (2022). Aptamer-modified M cell targeting liposomes for oral delivery of macromolecules. Colloids Surf. B Biointerfaces.

[154] Cui J., Wen Z., Zhang W., Wu W. (2022). Recent Advances in Oral Peptide or Protein-Based Drug Liposomes. Pharmaceuticals.

[155] Yun Y., Cho Y.W., Park K. (2013). Nanoparticles for oral delivery: Targeted nanoparticles with peptidic ligands for oral protein delivery. Adv. Drug Deliv. Rev..

[156] Wang A., Yang T., Fan W., Yang Y., Zhu Q., Guo S., Zhu C., Yuan Y., Zhang T., Gan Y. (2018). Protein Corona Liposomes Achieve Efficient Oral Insulin Delivery by Overcoming Mucus and Epithelial Barriers. Adv. Healthc. Mater..

[157] Ding R., Zhao Z., He J., Tao Y., Zhang H., Yuan R., Sun K., Shi Y. (2023). Preparation, Drug Distribution, and In Vivo Evaluation of the Safety of Protein Corona Liposomes for Liraglutide Delivery. Nanomaterials.

[158] Blanquet S., Marol-Bonnin S., Beyssac E., Pompon D., Renaud M., Alric M. (2001). The ‘biodrug’ concept: An innovative approach to therapy. Trends Biotechnol..

[159] Fei Z., Li S., Wang J., Wang Y., Jiang Z., Huang W., Sun H. (2019). *Rhodotorula glutinis* as a living cell liposome to deliver polypeptide drugs in vivo. Drug Deliv..

[160] Kimiz-Gebologlu I., Oncel S.S. (2022). Exosomes: Large-scale production, isolation, drug loading efficiency, and biodistribution and uptake. J. Control. Release.

[161] Zhong J., Xia B., Shan S., Zheng A., Zhang S., Chen J., Liang X.-J. (2021). High-quality milk exosomes as oral drug delivery system. Biomaterials.

[162] Wu L., Wang L., Liu X., Bai Y., Wu R., Li X., Mao Y., Zhang L., Zheng Y., Gong T. (2021). Milk-derived exosomes exhibit versatile effects for improved oral drug delivery. Acta Pharm. Sin. B.

[163] Xiao P., Wang H., Liu H., Yuan H., Guo C., Feng Y., Qi P., Yin T., Zhang Y., He H. (2024). Milk Exosome–Liposome Hybrid Vesicles with Self-Adapting Surface Properties Overcome the Sequential Absorption Barriers for Oral Delivery of Peptides. ACS Nano.

[164] Haddadzadegan S., To D., Matteo Jörgensen A., Wibel R., Laffleur F., Bernkop-Schnürch A. (2024). Comparative Analysis of PEG-Free and PEG-Based Self-Emulsifying Drug Delivery Systems for Enhanced Oral Bioavailability of Therapeutic (Poly) Peptides. Small.

[165] Mahmood A., Bernkop-Schnürch A. (2018). SEDDS: A game changing approach for the oral administration of hydrophilic macromolecular drugs. Adv. Drug Deliv. Rev..

[166] Menzel C., Holzeisen T., Laffleur F., Zaichik S., Abdulkarim M., Gumbleton M., Bernkop-Schnürch A. (2018). In vivo evaluation of an oral self-emulsifying drug delivery system (SEDDS) for exenatide. J. Control. Release.

[167] Phan T.N.Q., Ismail R., Le-Vinh B., Zaichik S., Laffleur F., Bernkop-Schnürch A. (2020). The Effect of Counterions in Hydrophobic Ion Pairs on Oral Bioavailability of Exenatide. ACS Biomater. Sci. Eng..

[168] Lu Y., Wu L., Lin M., Bao X., Zhong H., Ke P., Dai Q., Yang Q., Tang X., Xu W. (2023). Double layer spherical nanoparticles with hyaluronic acid coating to enhance oral delivery of exenatide in T2DM rats. Eur. J. Pharm. Biopharm..

[169] Maji I., Mahajan S., Sriram A., Medtiya P., Vasave R., Khatri D.K., Kumar R., Singh S.B., Madan J., Singh P.K. (2021). Solid self emulsifying drug delivery system: Superior mode for oral delivery of hydrophobic cargos. J. Control. Release.

[170] Sun X., Lv G., Xiong J., Zhao J., Zhao J., Wang Z., Wang Y., Yin T., Gou J., He H. (2024). Novel solid self-emulsifying drug delivery system to enhance oral bioavailability of cabazitaxel. Int. J. Pharm..

[171] Yuan H., Guo C., Liu L., Zhao L., Zhang Y., Yin T., He H., Gou J., Pan B., Tang X. (2023). Progress and prospects of polysaccharide-based nanocarriers for oral delivery of proteins/peptides. Carbohydr. Polym..

[172] Wang Y., Li H., Rasool A., Wang H., Manzoor R., Zhang G. (2024). Polymeric nanoparticles (PNPs) for oral delivery of insulin. J. Nanobiotechnol..

[173] Sarah L.C., Stephanie P.B., Lisa M., Jane K.P., Vitaliy V.K. (2017). Mucoadhesion: A food perspective. Food Hydrocoll..

[174] Yang J.-M., Wu L.-J., Lin M.-T., Lu Y.-Y., Wang T.-T., Han M., Zhang B., Xu D.-H. (2022). Construction and Evaluation of Chitosan-Based Nanoparticles for Oral Administration of Exenatide in Type 2 Diabetic Rats. Polymers.

[175] Sadeghi S., Lee W.K., Kong S.N., Shetty A., Drum C.L. (2020). Oral administration of protein nanoparticles: An emerging route to disease treatment. Pharmacol. Res..

[176] Bao X., Qian K., Xu M., Chen Y., Wang H., Pan T., Wang Z., Yao P., Lin L. (2023). Intestinal epithelium penetration of liraglutide via cholic acid pre-complexation and zein/rhamnolipids nanocomposite delivery. J. Nanobiotechnol..

[177] Li Q., Wen C., Yang J., Zhou X., Zhu Y., Zheng J., Cheng G., Bai J., Xu T., Ji J. (2022). Zwitterionic Biomaterials. Chem. Rev..

[178] Zhao Z., Ding R., Wang Y., Yuan R., Zhang H., Li T., Zheng W., Chen E., Wang A., Shi Y. (2024). Sulfobetaine modification of poly (D, L-lactide-co-glycolic acid) nanoparticles enhances mucus permeability and improves bioavailability of orally delivered liraglutide. J. Drug Deliv. Sci. Technol..

[179] Florek J., Caillard R., Kleitz F. (2017). Evaluation of mesoporous silica nanoparticles for oral drug delivery—Current status and perspective of MSNs drug carriers. Nanoscale.

[180] Abeer M.M., Rewatkar P., Qu Z., Talekar M., Kleitz F., Schmid R., Lindén M., Kumeria T., Popat A. (2020). Silica nanoparticles: A promising platform for enhanced oral delivery of macromolecules. J. Control. Release.

[181] Ehsan S., Mahdis M., Elahe M., Ahmad K., Mohammad Reza S., Zohreh S., Iman R., Shahriar D., Siyavash J., Iraj S. (2023). Exploring mesoporous silica nanoparticles as oral insulin carriers: In-silico and in vivo evaluation. Heliyon.

[182] Abeer M.M., Meka A.K., Pujara N., Kumeria T., Strounina E., Nunes R., Costa A., Sarmento B., Hasnain S.Z., Ross B.P. (2019). Rationally Designed Dendritic Silica Nanoparticles for Oral Delivery of Exenatide. Pharmaceutics.

[183] Xia J., Xue Y., Lei B., Xu L., Sun M., Li N., Zhao H., Wang M., Luo M., Zhang C. (2020). Multimodal channel cancer chemotherapy by 2D functional gadolinium metal–organic framework. Natl. Sci. Rev..

[184] Wei Q., Wu Y., Liu F., Cao J., Liu J. (2021). Advances in antitumor nanomedicine based on functional metal–organic frameworks beyond drug carriers. J. Mater. Chem. B.

[185] He L., Shang M., Chen Z., Yang Z. (2023). Metal-Organic Frameworks Nanocarriers for Functional Nucleic Acid Delivery in Biomedical Applications. Chem. Rec..

[186] Zou J.-J., Wei G., Xiong C., Yu Y., Li S., Hu L., Ma S., Tian J. (2022). Efficient oral insulin delivery enabled by transferrin-coated acid-resistant metal-organic framework nanoparticles. Sci. Adv..

[187] Agrawal A., Joshi A., Bhattacharya S. (2024). Recent Excavation of Nanoethosomes in Current Drug Delivery. Curr. Drug Deliv..

